# Supplementary material for: The DNA demethylase TET1 modifies the impact of maternal folic acid status on embryonic brain development
Source: EMBO Rep. 2024 Nov 22;26(1):175–99. doi: 10.1038/s44319-024-00316-1 (PMC11724065; doi:10.1038/s44319-024-00316-1)
Supplement: Supplementary file 1 — Table EV1 [file 44319_2024_316_MOESM1_ESM.pdf]

Table EV1 HPLC-MS/MS sample list

No.	TP	Group/Diet	Genotype	Phenotype	Theiler stage	Gender	Comments
1	TP15.6	30ppm+SST	WT	brain malformation	19/20	♀	
2	TP20.8	30ppm+SST	WT	normal	19/20	♀	
3	TP29.2	30ppm+SST	WT	normal	19/20	♀	
4	TP20.6	30ppm+SST	WT	brain malformation	19/20	♂	
5	TP25.6	30ppm+SST	WT	normal	19/20	♂	excluded in the heatmap analysis
6	TP29.5	30ppm+SST	WT	normal	19/20	♂	
7	TP20.5	30ppm+SST	HET	brain malformation	19/20	♀	
8	TP11.8	30ppm+SST	HET	normal	19/20	♀	
9	TP25.3	30ppm+SST	HET	normal	19/20	♀	
10	TP26.4	30ppm+SST	HET	brain malformation	19/20	♂	
11	TP11.3	30ppm+SST	HET	normal	19/20	♂	
12	TP15.7	30ppm+SST	HET	normal	19/20	♂	
13	TP11.7	30ppm+SST	KO	normal	19/20	♀	
14	TP15.4	30ppm+SST	KO	normal	19/20	♀	
15	TP20.1	30ppm+SST	KO	normal	19/20	♀	
16	TP20.2	30ppm+SST	KO	normal	19/20	♂	
17	TP26.2	30ppm+SST	KO	normal	19/20	♂	
18	TP29.1	30ppm+SST	KO	normal	19/20	♂	
19	TP18.4	3ppm+SST	WT	normal	20	♀	2♀, 4♂
20	TP24.1	3ppm+SST	WT	normal	19/20	♀	
21	TP24.2	3ppm+SST	WT	normal	19/20	♂	
22	TP27.1	3ppm+SST	WT	normal	19/20	♂	
23	TP27.2	3ppm+SST	WT	normal	19/20	♂	
24	TP33.8	3ppm+SST	WT	normal	19/20	♂	
25	TP12.3	3ppm+SST	HET	delayed	18	♀	excluded in the heatmap analysis
26	TP12.7	3ppm+SST	HET	normal	19/20	♀	
27	TP27.4	3ppm+SST	HET	normal	19/20	♀	
28	TP12.8	3ppm+SST	HET	normal	19/20	♂	
29	TP24.5	3ppm+SST	HET	normal	19/20	♂	
30	TP30.1	3ppm+SST	HET	normal	20	♂	
31	TP12.1	3ppm+SST	KO	NTD	19/20	♀	4♀, 2♂
32	TP30.2	3ppm+SST	KO	NTD	20	♀	
33	TP27.5	3ppm+SST	KO	normal	19/20	♀	
34	TP33.1	3ppm+SST	KO	normal	19/20	♀	
35	TP18.3	3ppm+SST	KO	normal	20	♂	
36	TP30.3	3ppm+SST	KO	normal	20	♂	
37	TP14.7	0.1ppm+SST	WT	normal	20	♀	
38	TP21.7	0.1ppm+SST	WT	normal	19/20	♀	
39	TP32.4	0.1ppm+SST	WT	normal	20	♀	
40	TP21.2	0.1ppm+SST	WT	brain malformation	19/20	♂	
41	TP21.6	0.1ppm+SST	WT	normal	19/20	♂	
42	TP23.6	0.1ppm+SST	WT	normal	19/20	♂	
43	TP16.5	0.1ppm+SST	HET	brain malformation	19	♀	
44	TP23.1	0.1ppm+SST	HET	NTD	19	♀	
45	TP28.4	0.1ppm+SST	HET	normal	19	♀	
46	TP16.7	0.1ppm+SST	HET	NTD	18	♂	
47	TP23.5	0.1ppm+SST	HET	brain malformation	19	♂	
48	TP14.2	0.1ppm+SST	HET	normal	20	♂	
49	TP14.5	0.1ppm+SST	KO	normal	20	♀	
50	TP21.4	0.1ppm+SST	KO	NTD, delayed	18/19	♀	
51	TP32.1	0.1ppm+SST	KO	normal	20	♀	
52	TP14.4	0.1ppm+SST	KO	normal	20	♂	
53	TP16.1	0.1ppm+SST	KO	normal	19	♂	
54	TP23.3	0.1ppm+SST	KO	normal	19	♂	

Table EV1. HPLC-MS/MS sample list

54 whole embryo samples were used for HPLC-MS/MS analysis. Embryos were first phenotyped and genotyped, and then Theiler stage- and sex-matched embryos were selected for mass spectrometry analysis. The genders of the samples were distributed equally across each genotype per diet group, unless otherwise indicated in the comment. Sample No.5 and 25 were outliers and excluded from the heatmap analysis.
