# Supplementary material for: The DNA demethylase TET1 modifies the impact of maternal folic acid status on embryonic brain development
Source: EMBO Rep. 2024 Nov 22;26(1):175–99. doi: 10.1038/s44319-024-00316-1 (PMC11724065; doi:10.1038/s44319-024-00316-1)
Supplement: Supplementary file 2 — Table EV2 [file 44319_2024_316_MOESM2_ESM.pdf]

Table EV2 RRBS sample list

No.	TP	Group/Diet	Genotype	Phenotype	Theiler stage	gender	Comments
1	36.3	30ppm + SST	WT	normal	19/20	♀	
2	41.3	30ppm + SST	WT	normal	19/20	♀	
3	43.7	30ppm + SST	WT	normal	19/20	♂	
4	36.6	30ppm + SST	HET	brain malformation	19/20	♀	
5	36.2	30ppm + SST	HET	normal	19/20	♀	
6	36.4	30ppm + SST	HET	brain malformation	19/20	♀	
7	38.3	30ppm + SST	HET	normal	19/20	♂	
8	38.7	30ppm + SST	HET	brain malformation	19/20	♂	
9	41.2	30ppm + SST	HET	normal	19/20	♂	
10	36.7	30ppm + SST	KO	NTD	19/20	♀	
11	38.6	30ppm + SST	KO	NTD	19/20	♂	
12	41.4	30ppm + SST	KO	brain malformation	19/20	♂	
13	41.8	30ppm + SST	KO	normal	19	♀	
14	43.4	30ppm + SST	KO	normal	19/20	♂	
15	40.2	3ppm + SST	WT	normal	19/20	♂	
16	40.3	3ppm + SST	WT	normal	19/20	♀	
17	45.3	3ppm + SST	WT	normal	19/20	♀	
18	57.3	3ppm + SST	WT	normal	19/20	♂	
19	40.1	3ppm + SST	HET	normal	19	♀	
20	42.1	3ppm + SST	HET	normal	19/20	♂	
21	45.6	3ppm + SST	HET	normal	19/20	♀	
22	40.10	3ppm + SST	KO	normal	19	♂	
23	40.5	3ppm + SST	KO	normal	19	♀	
24	42.9	3ppm + SST	KO	brain malformation	19/20	♂	
25	45.8	3ppm + SST	KO	normal	19/20	♀	
26	57.5	3ppm + SST	KO	NTD	19/20	♂	
27	34.3	0.1ppm + SST	WT	normal	19/20	♂	
28	50.1	0.1ppm + SST	WT	brain malformation	19/20	♀	
29	34.1	0.1ppm + SST	HET	normal	19/20	♂	
30	35.2	0.1ppm + SST	HET	normal	19/20	♀	excluded in the analysis
31	54.7	0.1ppm + SST	HET	normal	19	♂	
32	34.5	0.1ppm + SST	KO	normal	19/20	♂	
33	35.4	0.1ppm + SST	KO	brain malformation	19/20	♀	
34	50.3	0.1ppm + SST	KO	NTD	19/20	♀	
35	50.5	0.1ppm + SST	KO	NTD	19/20	♀	
36	54.8	0.1ppm + SST	KO	normal	19	♂	

Table EV2. RRBS sample list

36 embryonic brain tissues were used for RRBS analysis. Embryos were first phenotyped and genotyped, and then Theiler stage- and sex-matched embryos were selected for RRBS analysis. The genders of the samples were distributed equally across each genotype per diet group. Sample No.30 was outlier and excluded from data analysis.
