# Supplementary material for: The DNA demethylase TET1 modifies the impact of maternal folic acid status on embryonic brain development
Source: EMBO Rep. 2024 Nov 22;26(1):175–99. doi: 10.1038/s44319-024-00316-1 (PMC11724065; doi:10.1038/s44319-024-00316-1)
Supplement: Supplementary file 3 — Table EV3 [file 44319_2024_316_MOESM3_ESM.pdf]

Table EV3 Bulk RNA-seq sample list

No.	TP	Group/Diet	Genotype	Phenotype	Theiler stage	Gender	RIN	Comments
1	38.1	30ppm + SST	WT	normal	19/20	♀	10	
2	43.2	30ppm + SST	WT	normal	19/20	♀	10	
3	46.3	30ppm + SST	WT	grossly malformed	19/20	♂	9,2	excluded in the analysis
4	47.1	30ppm + SST	WT	normal	19/20	♂	10	
5	49.1	30ppm + SST	WT	normal	19/20	♀	10	
6	38.4	30ppm + SST	HET	brain malformation	19/20	♀	10	
7	43.3	30ppm + SST	HET	normal	19/20	♀	10	
8	46.7	30ppm + SST	HET	normal	19/20	♂	10	
9	46.8	30ppm + SST	HET	brain malformation	19/20	♂	10	
10	47.5	30ppm + SST	HET	normal	19/20	♀	10	
11	43.6	30ppm + SST	KO	normal	19/20	♂	10	
12	46.4	30ppm + SST	KO	grossly malformed	19/20	♂	9,1	excluded in the analysis
13	47.3	30ppm + SST	KO	NTD	19	♀	10	
14	47.6	30ppm + SST	KO	normal	19/20	♀	10	
15	49.2	30ppm + SST	KO	normal	19/20	♀	10	
16	51.9	3ppm + SST	WT	normal	20	♀	10	
17	53.2	3ppm + SST	WT	normal	19/20	♂	10	
18	57.8	3ppm + SST	WT	normal	19/20	♂	10	
19	45.4	3ppm + SST	HET	normal	19/20	♂	10	
20	51.2	3ppm + SST	HET	normal	20	♀	10	
21	53.4	3ppm + SST	HET	normal	19/20	♂	10	
22	45.1	3ppm + SST	KO	normal	19/20	♂	10	
23	51.6	3ppm + SST	KO	normal	20	♀	10	
24	53.1	3ppm + SST	KO	normal	19/20	♂	10	
25	57.6	3ppm + SST	KO	NTD	19/20	♂	10	
26	52.1	0.1ppm + SST	WT	normal	19	♀	9,9	
27	52.2	0.1ppm + SST	WT	normal	19	♂	10	
28	52.3	0.1ppm + SST	WT	brain malformation	19	♀	9,9	
29	34.2	0.1ppm + SST	HET	normal	19/20	♀	10	
30	34.4	0.1ppm + SST	HET	brain malformation	19/20	♀	9,9	
31	35.1	0.1ppm + SST	HET	normal	19/20	♂	10	
32	48.5	0.1ppm + SST	HET	normal	19/20	♂	10	
33	54.2	0.1ppm + SST	HET	normal	19/20	♂	10	
34	54.3	0.1ppm + SST	HET	brain malformation	19/20	♂	9,8	
35	34.7	0.1ppm + SST	KO	normal	19	♀	10	
36	35.5	0.1ppm + SST	KO	normal	19/20	♂	10	
37	48.1	0.1ppm + SST	KO	normal	19/20	♂	10	
38	50.6	0.1ppm + SST	KO	NTD	19/20	♀	9,6	
39	52.5	0.1ppm + SST	KO	delayed	18	♂	10	
40	54.1	0.1ppm + SST	KO	normal	19/20	♂	9,7	

Table EV3. Bulk RNA-seq sample list

40 embryonic brain tissues were used for RNA-seq analysis. Embryos were first phenotyped and genotyped, and then Theiler stage- and sex-matched embryos were selected for RNA-seq analysis. The genders of the samples were distributed equally across each genotype per diet group. Sample No.3 and 12 were outliers and excluded from data analysis.
