# Supplementary material for: The DNA demethylase TET1 modifies the impact of maternal folic acid status on embryonic brain development
Source: EMBO Rep. 2024 Nov 22;26(1):175–99. doi: 10.1038/s44319-024-00316-1 (PMC11724065; doi:10.1038/s44319-024-00316-1)
Supplement: Supplementary file 4 — Appendix [file 44319_2024_316_MOESM4_ESM.pdf]

Appendix

The DNA demethylase TET1 modifies the impact of maternal folic acid status on embryonic brain development

Lehua Chen[1, †], Bernard K. van der Veer[1, †], Qiuying Chen[3], Spyridon Champeris Tsaniras[1], Wannes Brangers[1], Harm H.M. Kwak[1], Rita Khoueiry[1], Yunping Lei[2], Robert Cabrera[2], Steven S. Gross[3], Richard H. Finnell[2, 4], Kian Peng Koh[1, 2, #]

† These authors contributed equally
# To whom correspondence should be addressed. Email: kian.koh@kuleuven.be or kian.koh@bcm.edu

Table of Contents:
Appendix Figure S1 .... Page 1
Appendix Figure S2 .... Page 4
Appendix Figure S3 .... Page 6
Appendix Figure S4 .... Page 8
Appendix Figure S5 .... Page 10
Appendix Figure S6 .... Page 12
Appendix Figure S7 .... Page 14
Appendix Figure S8 .... Page 16

Chen & van der Veer et al. Appendix Figure S1

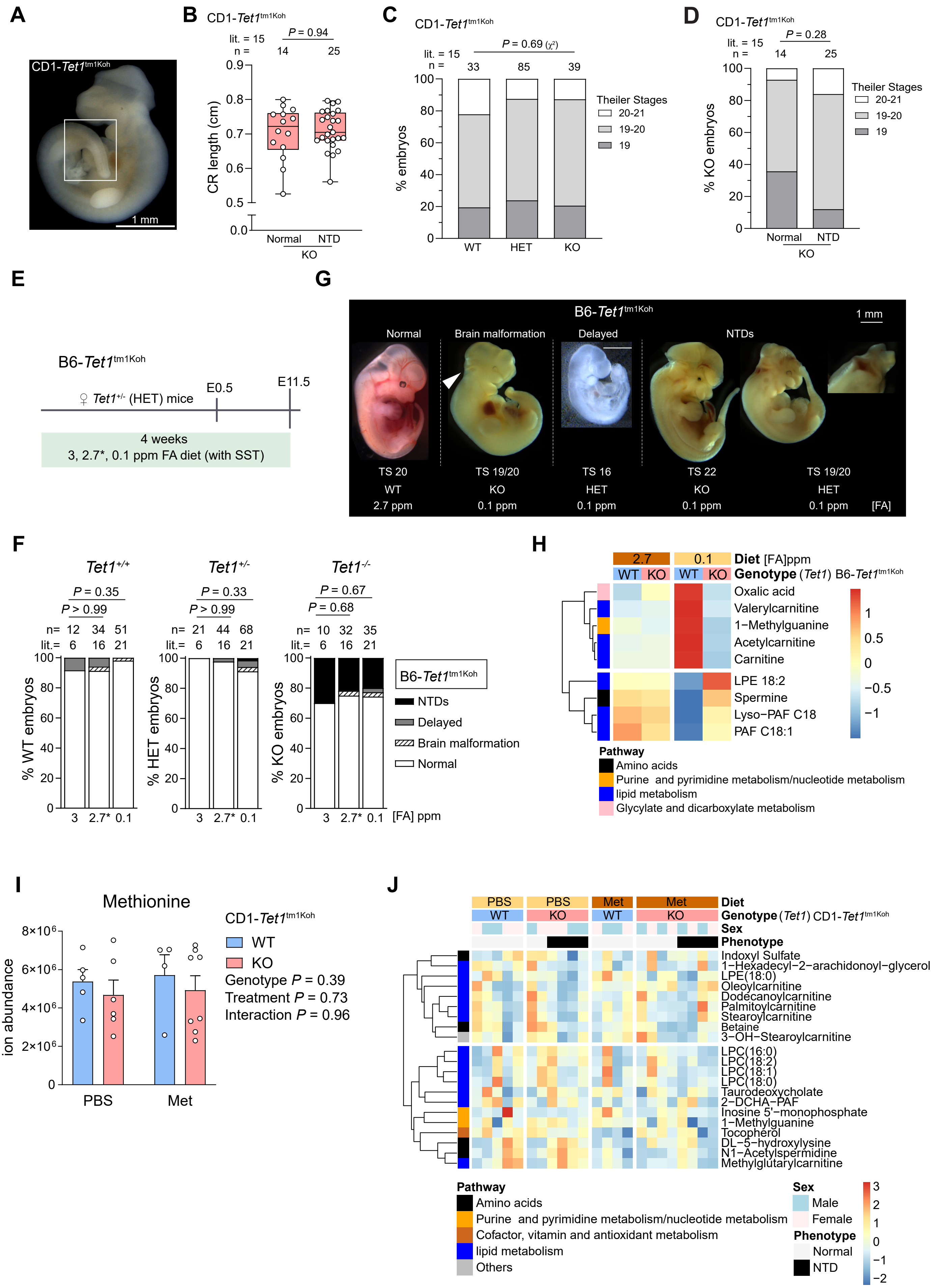

Appendix Figure S1. Phenotypic and metabolomic analysis of strains of *Tet1*$^{-/-}$ mice displaying high or low NTD penetrance and the characterization of strain responsiveness to FA and methionine supplementation (related to main Fig. 1).

(**A**) A lateral image of an E10.5 *Tet1*$^{-/-}$ embryo from the CD1-*Tet1*^{tm1Koh} outbred stock with cranial NTD. The tailbud is boxed to show complete closure of posterior neuropore. Scale bar is 1 mm. (**B**) Crown-rump (CR) length of CD1-*Tet1*^{tm1Koh} stock KO embryos classified by normal and NTD phenotype presentation. litter (lit.) = number of litters collected, n = number of embryos. *P* values are calculated by t-test. (**C**) Scoring of Theiler stages in CD1-*Tet1*^{tm1Koh} *Tet1*$^{+/+}$ (WT), *Tet1*$^{+/-}$ (HET) and *Tet1*$^{-/-}$ (KO) embryos. Overall *P* values are calculated by Chi square test. (**D**) Scoring of Theiler stages in CD1-*Tet1*^{tm1Koh} KO embryos classified by normal and NTD phenotypes. *P* values are calculated by Fisher's Exact test. (**E**) Schematic representation of the experimental design for FA depletion in the B6-*Tet1*^{tm1Koh} mouse strain. *, The custom 2.7 ppm FA diet (TestDiet® 5BRK) was composed of modified AIN-93G mineral mix (TestDiet® 57W5) supplemented with 0.19% inositol and 1% succinyl sulfathiazole (SST) antibiotic. 3 ppm and 0.1 ppm FA custom diets were subsequently made based on the same formulation as 5S5U (modified TestDiet® 57W5 with no added folate or inositol, supplemented with 1% SST). (**F**) Phenotypic scoring of *Tet1*$^{+/+}$, *Tet1*$^{+/-}$ and *Tet1*$^{-/-}$ embryos from the B6-*Tet1*^{tm1Koh} strain fed custom modified regular (3 ppm and 2.7 ppm) and depleted (0.1 ppm) FA maternal diets. *P* values are calculated by Fisher's Exact test. *P* values indicate the statistical comparison of all affected embryos with normal embryos in the *Tet1*$^{+/+}$ and *Tet1*$^{+/-}$ genotype groups, and of NTD-affected embryos with normal embryos in *Tet1*$^{-/-}$. (**G**) Representative images of embryos with distinct phenotypes in the B6-*Tet1*^{tm1Koh} strain. *Tet1* genotype and custom diet FA levels are indicated below each embryo. Arrow indicates the malformation in the midbrain-hindbrain juncture. The inset is the back view of the brain, indicating the NTD phenotype in the HET embryo. Scale bar is 1 mm. (**H**) Heatmap of metabolite ion abundances detectable by HPLC-MS/MS in E11.5 whole embryos of the B6-*Tet1*^{tm1Koh} strain WT and KO fed regular and depleted FA diets, showing only metabolites that displayed significant changes based on two-way ANOVA (overall $P < 0.05$) and in common with the list of significant metabolites shown in main Fig. 1I for CD1-*Tet1*^{tm1Koh} embryos supplemented with FA. n=4, WT embryos in 2.7 ppm group; n=5, WT embryos in 0.1ppm group; n=8, KO embryos in 2.7 ppm group; and n=5, KO embryos in 0.1ppm group. Overall *P* values are calculated by two-way ANOVA to compare the effects of genotype factor, treatment factor and genotype x treatment interaction, followed with a post-hoc Tukey's test to correct for multiple comparisons. (**I**) Detection of methionine (Met) in E11.5 CD1-*Tet1*^{tm1Koh} whole embryos following IP injections of 70 mg/kg methionine or phosphate-buffered saline (PBS) in pregnant dams. n=5, WT embryos in PBS group; n=4, WT embryos in Met

abundances in E11.5 CD1-*Tet1*^{tm1Koh} stock whole embryos treated by methionine or PBS injection. None of the compounds shown here displayed any significant changes with the treatment but are from the list of significant compounds shown in main Fig. 1I for CD1-*Tet1*^{tm1Koh} embryos supplemented with FA. n=5, WT embryos in PBS group; n=4, WT embryos in Met group; n=6, KO embryos in PBS group; and n=8, KO embryos in Met group.

A

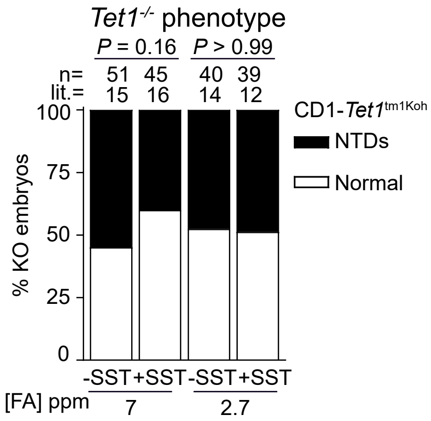

B

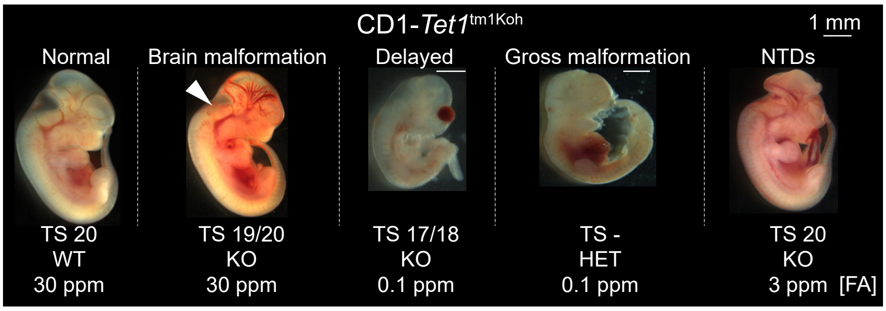

C

129S6.Cg-*Tet1*tm1Koh

	TP set up	TP failed	Plugged but not pregnant	Decidua	Embryos	Resorption	Resorption rates
30ppm	21	4	3	130	121	9	6,9
3ppm	23	11	6	104	87	17	16,3
0.1ppm	24	6	2	139	108	31	22,3
regular chow	17	6	5	90	83	7	7,8

D

129S6.Cg-*Tet1*tm1Koh

	[FA] ppm	NTDs	Gross malformation	Delayed	Brain malformation	Normal	Total
Tet1 $^{+/+}$	30	0	2	2	4	26	34
	3	0	2	0	1	22	25
	0.1	0	3	0	5	19	27
	regular chow	0	0	0	1	22	23
Tet1 $^{+/-}$	30	0	3	1	13	38	55
	3	0	1	1	3	35	40
	0.1	3	4	2	12	25	46
	regular chow	0	0	0	1	37	38
Tet1 $^{-/-}$	30	3	1	1	2	26	33
	3	4	0	0	1	16	21
	0.1	6	4	3	2	21	36
	regular chow	4	0	1	2	14	21

Appendix Figure S2. Phenotypic changes of CD1 and 129S6.Cg-*Tet1*$^{\mathrm{tm1Koh}}$ embryos in response to maternal dietary FA excess and depletion (related to main Fig. 2).

(**A**) Rates of NTDs in the CD1-*Tet1*$^{\mathrm{tm1Koh}}$ stock *Tet1*$^{-/-}$ embryos, in response to modified maternal diet containing either 7 or 2.7 ppm FA with or without SST. *P* values are calculated by Fisher's exact test. (**B**) Representative images of CD1-*Tet1*$^{\mathrm{tm1Koh}}$ stock embryos showing phenotypes associated with *Tet1* genotype and modified FA diet. Arrow indicates the malformation in the midbrain-hindbrain juncture. Scale bar is 1 mm. (**C**) Summary table of embryo resorption rates in timed pregnancies (TP) set up using 129S6.Cg-*Tet1*$^{\mathrm{tm1Koh}}$ dams adapted to the three FA-modified custom diets and regular chow. Plugged but not pregnant means that vaginal plug was detected but no pregnancy resulted at the E11.5 end-point. TP failed means that there was no successful mating based on absence of plug and pregnancy. (**D**) Tabulation of embryo counts by phenotypes observed in the 129S6.B6-*Tet1*$^{\mathrm{tm1Koh}}$ strain WT, HET and KO embryos, exposed to FA-modified maternal diet. The regular chow contains 7 ppm FA without SST.

Chen & van der Veer et al. Appendix Figure S3

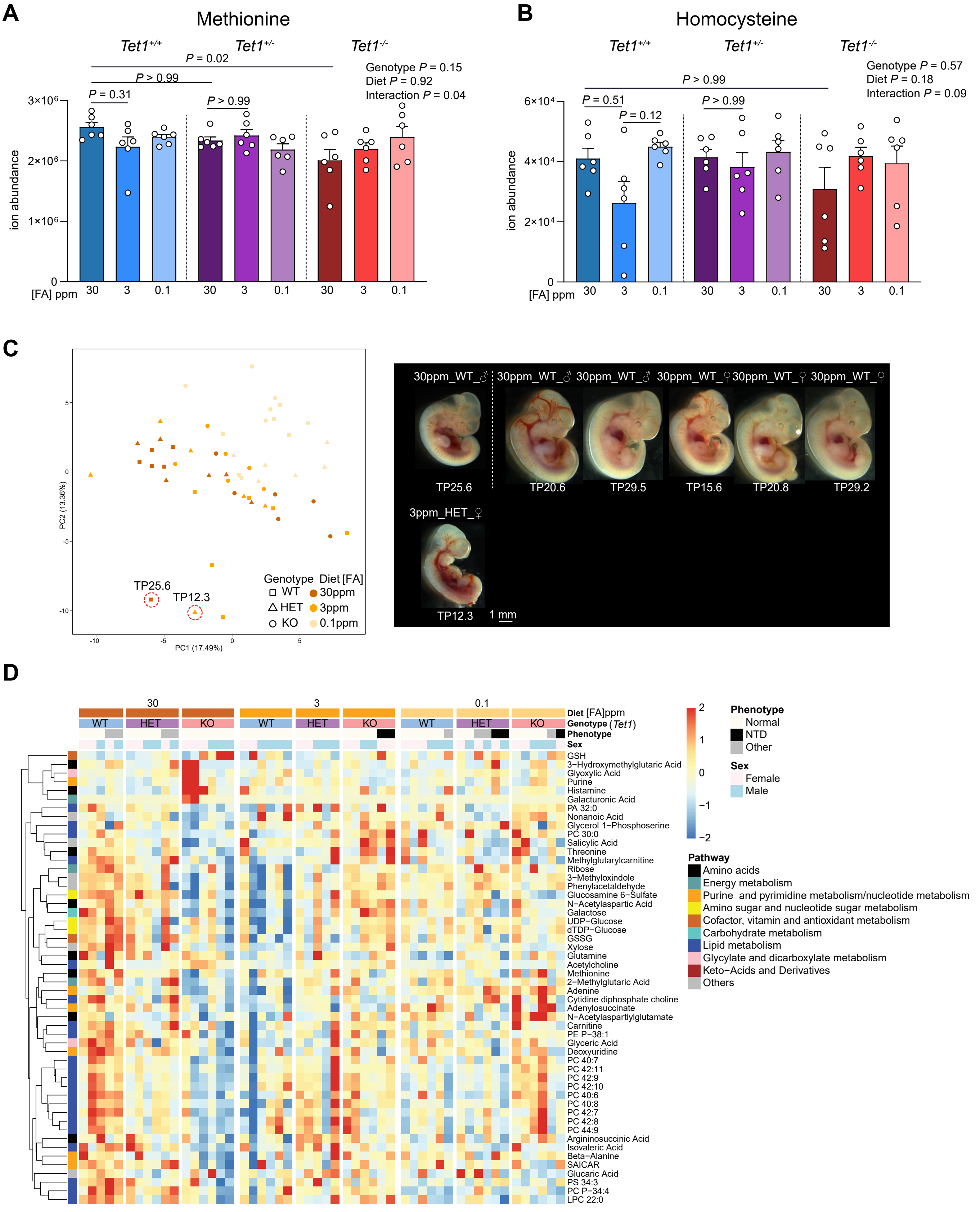

Appendix Figure S3. Metabolomic changes in 129S6.Cg embryos as an effect of *Tet1* genotype and modified maternal dietary FA status (related to main Fig. 3).

(**A**) and (**B**) HPLC-MS/MS ion abundance detection of methionine (A) and homocysteine (B) in whole embryos. Data are mean ± SEM from n=6 individual embryos, 3 males and 3 females per group, except WT in 30 ppm FA and KO in 3 ppm FA groups which consisted of 4 females and 2 males. Overall *P* values are calculated by two-way ANOVA followed with a post-doc Tukey's test for multiple comparison correction to show the significance of genotype factor, diet factor, and genotype x diet interaction. *P* values shown in pairwise comparisons were determined using one-way ANOVA and a post hoc analysis with the Dunn's multiple comparison test. (**C**) Principal component analysis of metabolomic data from individual E11.5 whole embryos. Two outliners in red circle are excluded in downstream 2-way ANOVA interaction analysis. Embryo images of outliners are shown in the right panel. TP25.6_30ppm_WT_male is a smaller embryo size compared to littermates shown to the right of the dashed line, while TP12.3_3ppm_HET_female is slightly deformed. (**D**) Expanded heatmap of main Fig. 3G, showing individual embryo per genotype per diet group with details of phenotype and sex in the 129S6.B6-*Tet1*^{tm1Koh} strain. 2-way ANOVA interaction $P < 0.05$. n=6 per genotype per diet, except n=5 in the two groups of HET in 3 ppm FA and WT in 30 ppm FA.

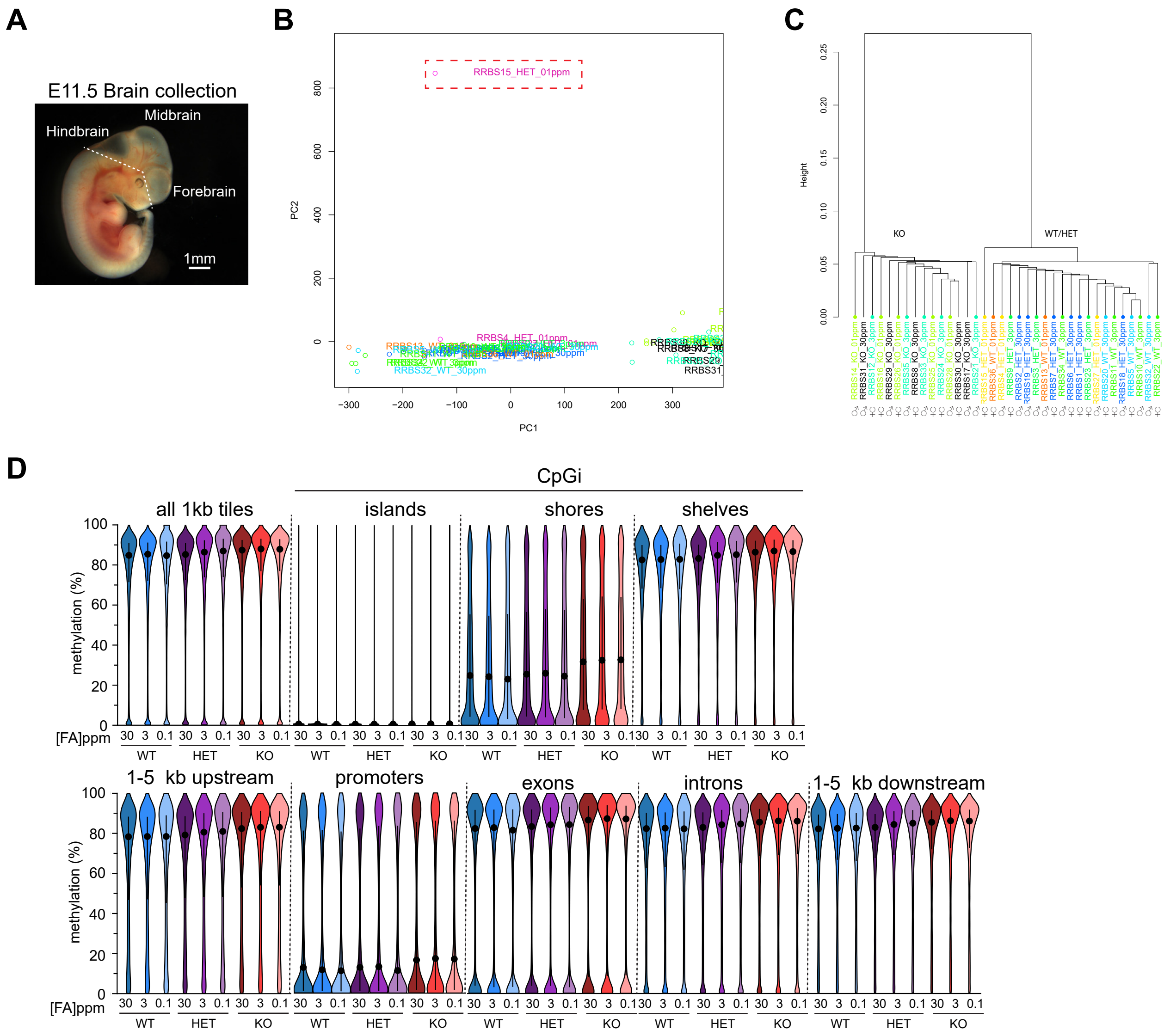
A
E11.5 Brain collection
Hindbrain
Midbrain
Forebrain
1mm
B
RRBS15_HET_01ppm
PC2
PC1
C
Height
KO
WT/HET
D
CpGi
all 1kb tiles
islands
shores
shelves
methylation (%)
[FA]ppm
WT
HET
KO
1-5 kb upstream
promoters
exons
introns
1-5 kb downstream

Appendix Figure S4. DNA methylation changes in 129S6.Cg E11.5 embryonic brains as an effect of *Tet1* genotype and maternal dietary FA excess or depletion (related to main Fig. 4).

(**A**) Schematic diagram of E11.5 embryonic brain dissection. Plan of dissection is shown as dotted line, above which embryonic brain tissues were collected for RRBS and RNA-seq analyses. (**B**) Principal component analysis of RRBS data from individual E11.5 embryonic brain samples. One outliner was excluded in downstream analysis because of poor mapping coverage. (**C**) Hierarchical clustering plot of individual samples after exclusion of one outliner. Left, cluster of KO samples; right, cluster of WT and HET samples. n=5, KO per diet group, matched to n=3 WT and n=6 HET in 30 ppm FA, n=4 WT and n=3 HET in 3 ppm FA, and n=2 WT and n=3 HET in 0.1 ppm FA. (**D**) Violin plot of methylation levels of 1 kb tiles across the genome and annotated gene features of all sample groups by FA diet and *Tet1* genotype.

Chen & van der Veer et al. Appendix Figure S5

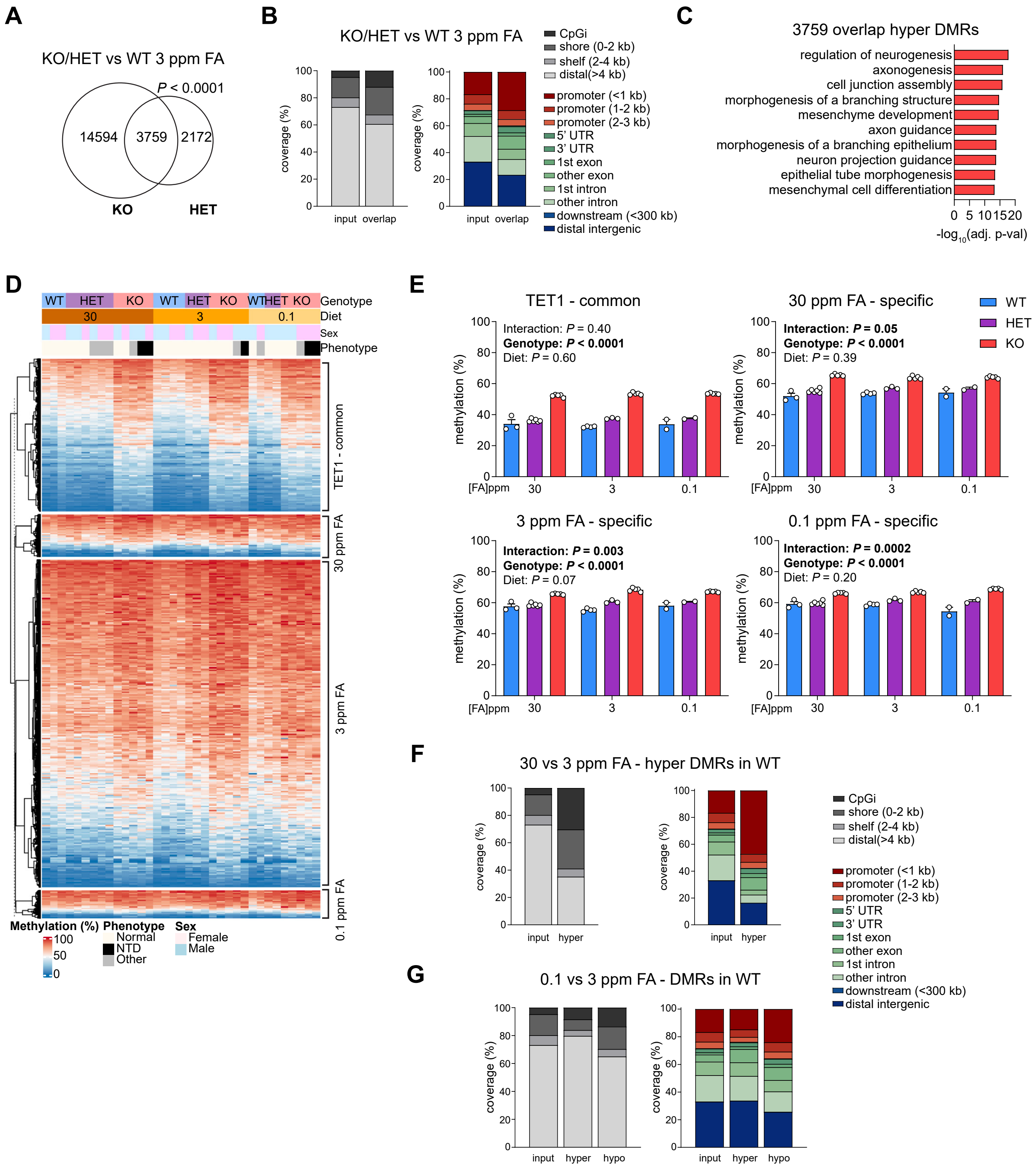

Appendix Figure S5. Analysis of differentially methylated regions (DMRs) in 129S6.Cg E11.5 embryonic brains as an effect of *Tet1* genotype and maternal dietary FA excess or depletion (related to main Fig. 4).

(**A**) Venn diagram of hyper DMRs defined by pairwise comparison of KO or HET vs WT in 3 ppm FA diet. (**B**) Distribution by CpG island (CpGi) proximity (left) and gene feature annotation (right) of 3759 hyper DMRs in the overlap shown in (A). (**C**) GO analysis of the 3759 overlapping hyper DMRs. (**D**) Expanded heatmap of methylation levels of hyper DMRs classified as "TET1 common" in all three custom FA diet groups, or as "TET1 x FA diet- specific" to each diet group, showing individual embryo per genotype per diet group annotated for phenotype and sex. (**E**) Methylation levels at hyper DMRs in KO vs WT classified as common in all diets or specific to each diet across all groups. Overall *P* values are calculated by two-way ANOVA to compare the effect of genotype factor, diet factor, and genotype x diet interaction, followed by a post-hoc Tukey's test for multiple comparison correction. (**F**) Distribution by CpGi proximity (left) and gene feature annotation (right) of the 857 hyper DMRs induced by FA excess in WT embryos as shown in main Fig. 4G (left). (**G**) Distribution by CpGi proximity (left) and gene feature annotation (right) of 2275 hyper and 2969 hypo DMRs induced by FA depletion in WT embryos as shown in main Fig. 4G (right).

Chen & van der Veer et al. Appendix Figure S6

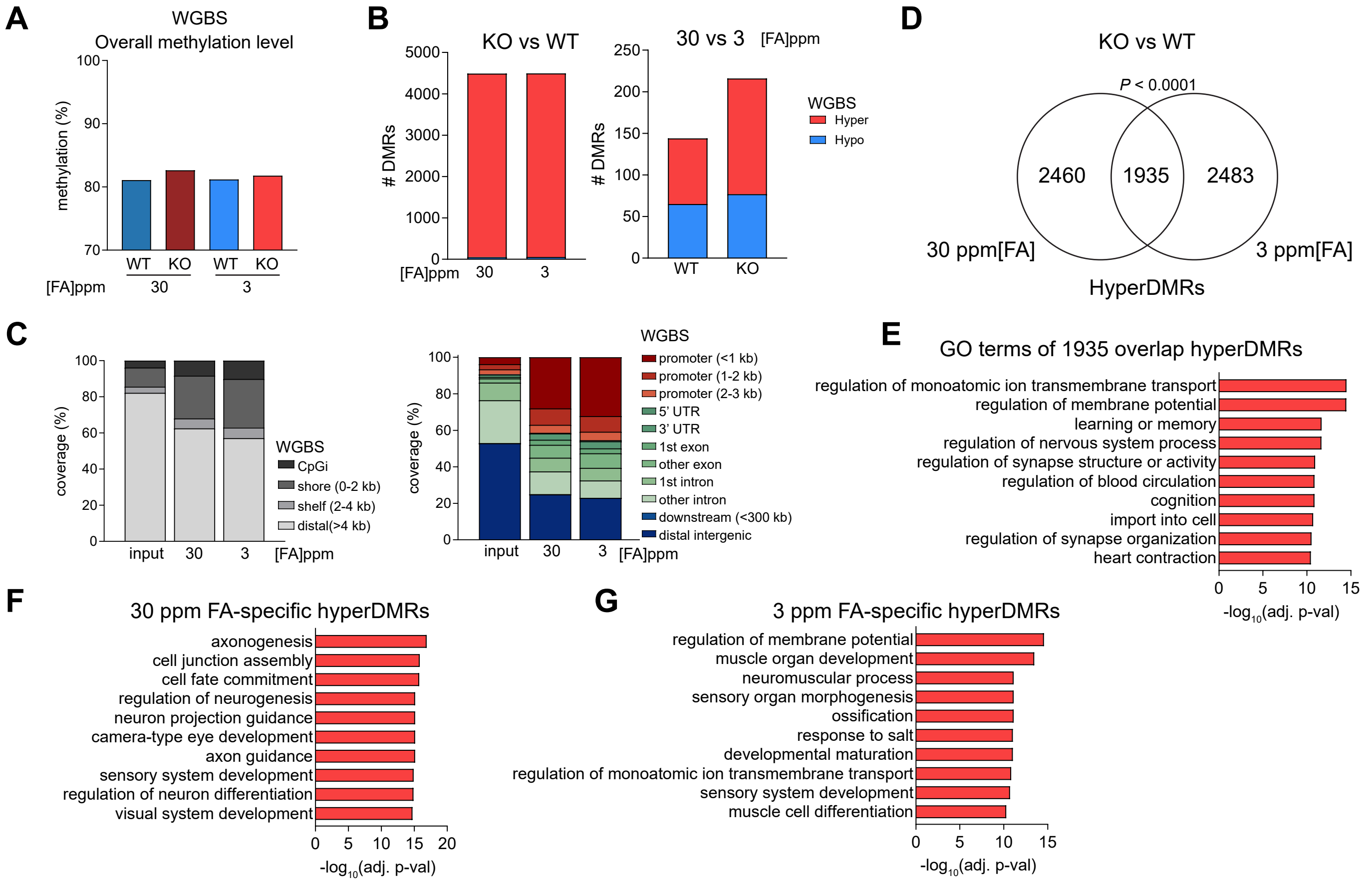

Appendix Figure S6. Whole-genome DNA methylome analysis of 129S6.Cg E11.5 WT and *Tet1* KO embryonic brains exposed to regular or excess maternal dietary FA (related to main Fig. 4).

(**A**) Global CpG methylation levels per sample. (**B**) Number of hyper and hypo DMRs defined by pairwise comparisons of *Tet1* KO vs WT per diet (left) and by 30 vs 3 ppm FA diet per *Tet1* genotype (right). (**C**) Distribution by CpGi proximity (left) and gene feature annotation (right) of KO vs WT hyper DMRs per diet. (**D**) Venn diagram of KO vs WT hyper DMRs in the two diet groups. (**E** - **G**) GO analysis of 1935 hyper DMRs in the Venn overlap (E), 2460 in the non-overlap of 30 ppm FA diet (F), and 2483 in the non-overlap of the 3 ppm FA diet group (G) as defined in (D).

Chen & van der Veer et al. Appendix Figure S7

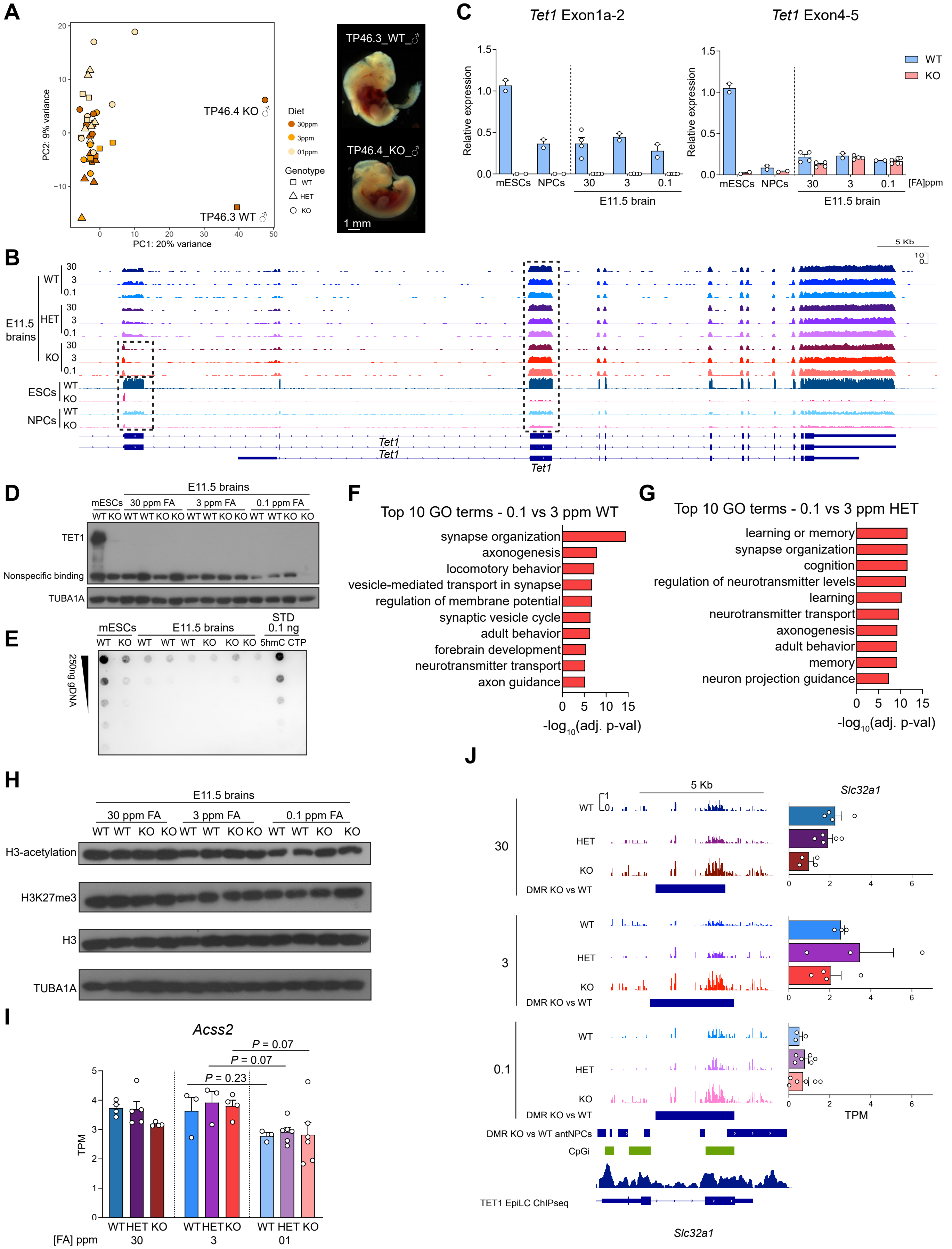

Appendix Figure S7. Gene expression changes in 129S6.Cg E11.5 embryonic brains as an effect of *Tet1* genotype and maternal dietary FA excess or depletion (related to main Fig. 5).

(**A**) PCA of RNA-seq data from individual E11.5 embryonic brain samples. Two outliners are marked on the plot and excluded in downstream analysis. Embryo images of outliners are shown in the right panel. (**B**) IGV tracks of RNA-seq signals over the *Tet1* gene locus in E11.5 WT, HET, and KO embryonic brains per FA diet group, and in WT and KO ESCs and *in vitro* differentiated antNPCs (van der Veer et al. 2023). Dashed box on the left denotes ablation of transcripts in the 5' coding exon (exon 2) targeted by the knockin-knockout construct. Dashed box over a downstream exon on the right indicates expression of a short *Tet1* isoform in the E11.5 KO brains from a downstream TSS. Annotations of the embryonic full-length *Tet1* and the short (somatic) *Tet1* transcript isoforms are indicated in the bottom panel. (**C**) qPCR analysis of *Tet1* detected by primers spanning exons 1a-2 and exons 3-4 in WT and KO ESCs, *in vitro* differentiated NPCs, and E11.5 WT and KO embryonic brains per custom diet group, verifying the RNA-seq *Tet1* transcript levels as shown in (B). (**D**) Western blot of TET1 in WT and KO ESCs and E11.5 embryonic brains per custom diet group. TUBA1A, alpha-tubulin loading control. (**E**) DNA dot blot of 5hmC in WT and KO ESCs and E11.5 brains. 5hmC and CTP are positive and negative control standards (STD), respectively. (**F**) and (**G**) Top 10 GO terms associated with 373 and 777 downregulated DEGs defined by pairwise comparison of 0.1 ppm vs 3 ppm FA diet in WT and HET genotypes, respectively, as shown in main Fig. 5E (right panel). (**H**) Western blot of pan histone H3 acetylation and H3K27me3 in the 129S6.Cg-*Tet1*^{tm1Koh} strain KO and WT E11.5 brains in the three FA diet groups. TUBA1A, alpha-tubulin loading control. (**I**) RNA-seq normalized gene expression in transcript per million (TPM) of *Acss2* in WT, HET, and KO brains per diet group. *P* values are calculated by one-way ANOVA. (**J**) IGV tracks of RRBS CpG methylation levels over a DMR at *Slc32a1* in WT, HET, and KO brains per diet group (left). Locations of DMRs identified from WGBS of KO vs WT antNPCs (van der Veer et al. 2023), CpGi annotation and TET1 ChIP-seq signals in EpiLC (Khoueiry et al. 2017) are indicated in the bottom panel. RNA-seq TPM expression of *Slc32a1* per group is shown on the right.

Chen & van der Veer et al. Appendix Figure S8

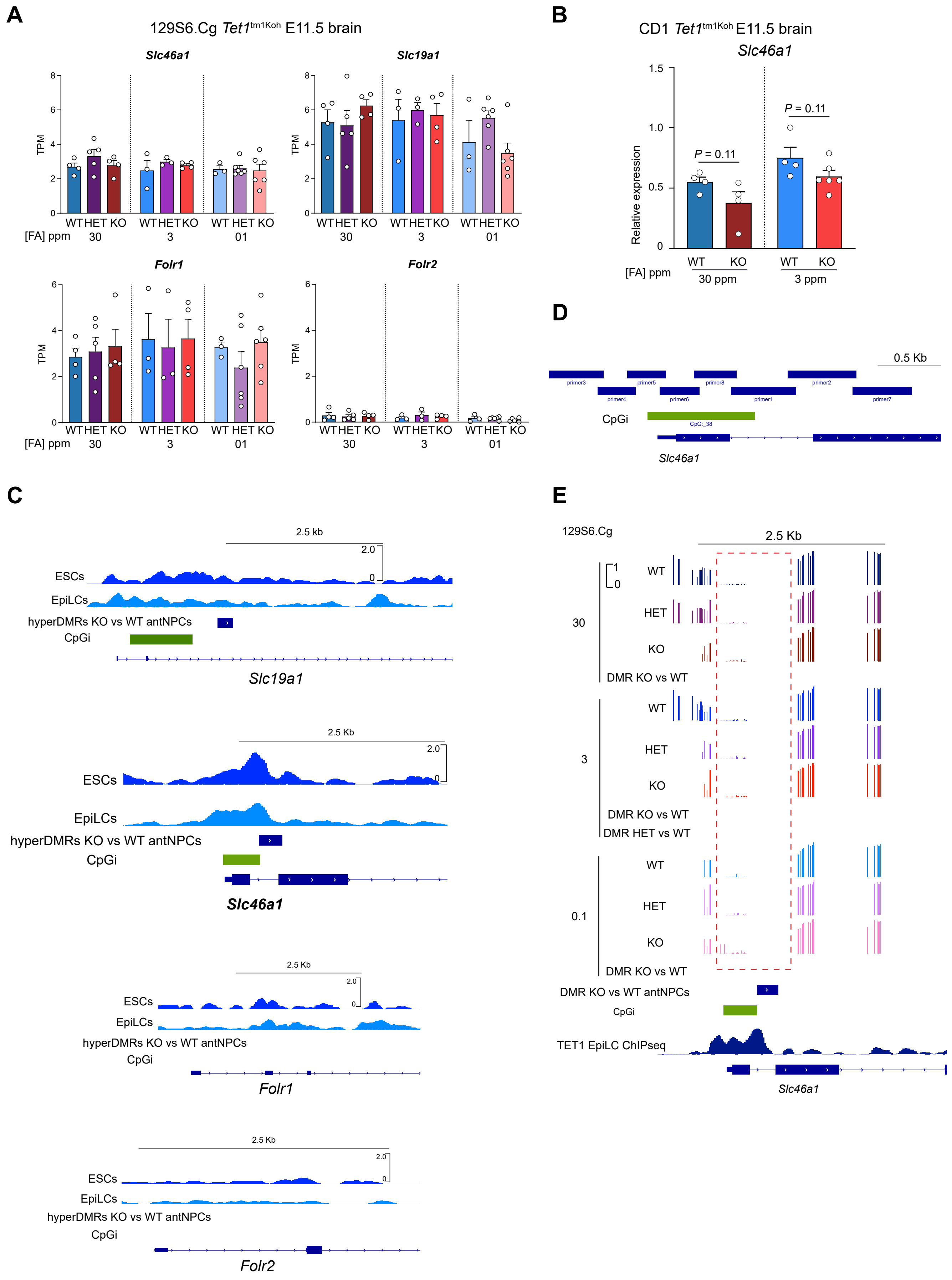

Appendix Figure S8. Gene expression and DNA methylation analysis of folate transporters (related to main Fig. 6).

(**A**) RNA-seq gene expression of folate transporters *Slc46a1* and *Slc19a1* (top row), folate receptors *Folr1* and *Folr2* (bottom row) in 129S6.Cg E11.5 embryonic brains by *Tet1* genotype and diet groups**.** (**B**) qPCR analysis of *Slc46a1* in E11.5 brains exposed to 30 ppm and 3 ppm FA in CD1-*Tet1*^{tm1Koh} mice. n = 6, KO in 3 ppm FA and n=4, all other groups. *P* values are calculated by Student's t-test. (**C**) IGV snapshots of TET1 ChIP-seq tracks in mESCs and EpiLCs (Luo et al. 2020), illustrating the occupancy of *Tet1* on the promoter regions of *Slc19a1*, *Slc46a1*, *Folr1*, and *Folr2*. HyperDMRs are defined by pairwise comparison of WGBS data of *Tet1* KO and WT antNPCs of a (B6x129S6)F1 strain differentiated *in vitro* from ESCs in the presence of a Wnt inhibitor (day 5 + XAV) (**D**) Overview of primer amplicons designed to cover the promoter region (~1.3 Mb) of *Slc46a1*. (**E**) IGV tracks of RRBS CpG methylation levels over the *Slc46a1* promoter region in WT, HET, and KO brains per FA diet (top), indicating a CpGi shore region with no coverage by RRBS. Location of a DMR from WGBS of KO vs WT antNPCs (van der Veer et al. 2023), CpGi annotation and TET1 ChIP-seq signals in EpiLC (Khoueiry et al. 2017), are indicated in the bottom panel. The red dashed box highlights the region with no coverage by RRBS.
