# Supplementary figures and images for: The DNA demethylase TET1 modifies the impact of maternal folic acid status on embryonic brain development
Source: EMBO Rep. 2024 Nov 22;26(1):175–99. doi: 10.1038/s44319-024-00316-1 (PMC11724065; doi:10.1038/s44319-024-00316-1)

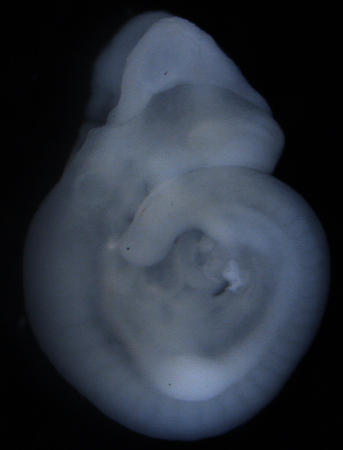

Supplement: Supplementary file 6 — Source data Fig. 1 [file 44319_2024_316_MOESM6_ESM.zip › Soure data_Figure 1/Figure 1B/E10.5/TP42.2(ko).tif]

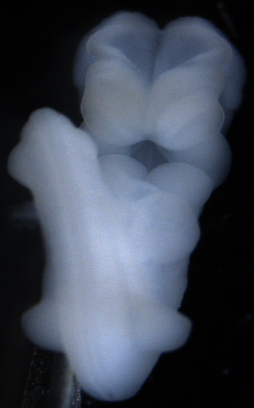

Supplement: Supplementary file 6 — Source data Fig. 1 [file 44319_2024_316_MOESM6_ESM.zip › Soure data_Figure 1/Figure 1B/E10.5/TP42.2(ko)front.tif]

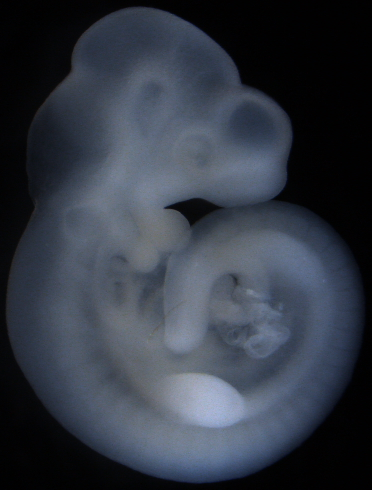

Supplement: Supplementary file 6 — Source data Fig. 1 [file 44319_2024_316_MOESM6_ESM.zip › Soure data_Figure 1/Figure 1B/E10.5/TP42.3(het).tif]

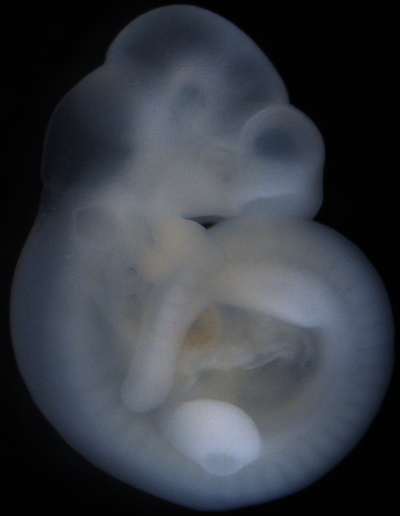

Supplement: Supplementary file 6 — Source data Fig. 1 [file 44319_2024_316_MOESM6_ESM.zip › Soure data_Figure 1/Figure 1B/E10.5/TP42.5(wt).tif]

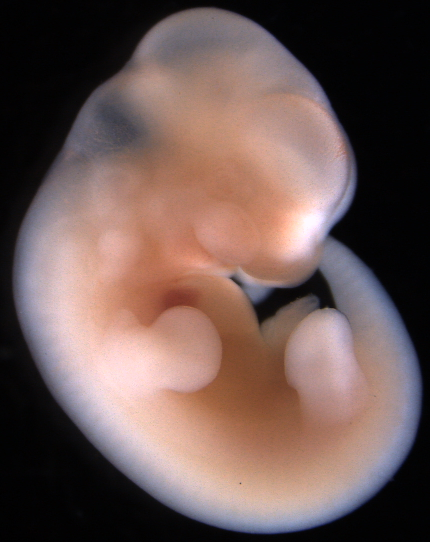

Supplement: Supplementary file 6 — Source data Fig. 1 [file 44319_2024_316_MOESM6_ESM.zip › Soure data_Figure 1/Figure 1B/E11.5/TP46.10(wt).tif]

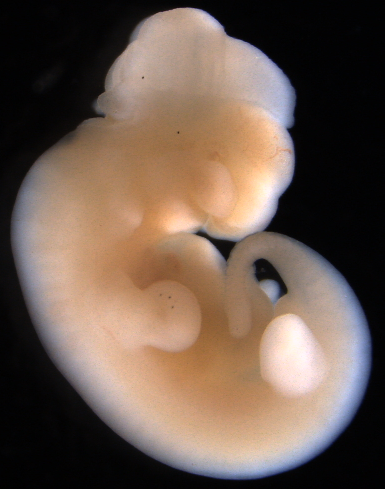

Supplement: Supplementary file 6 — Source data Fig. 1 [file 44319_2024_316_MOESM6_ESM.zip › Soure data_Figure 1/Figure 1B/E11.5/TP46.12(ko).tif]

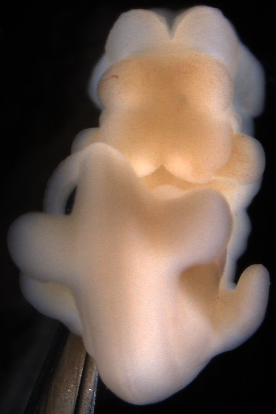

Supplement: Supplementary file 6 — Source data Fig. 1 [file 44319_2024_316_MOESM6_ESM.zip › Soure data_Figure 1/Figure 1B/E11.5/TP46.12(ko)front.tif]

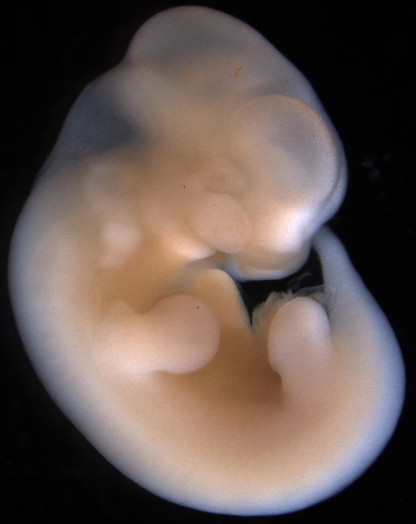

Supplement: Supplementary file 6 — Source data Fig. 1 [file 44319_2024_316_MOESM6_ESM.zip › Soure data_Figure 1/Figure 1B/E11.5/TP46.6(het).tif]

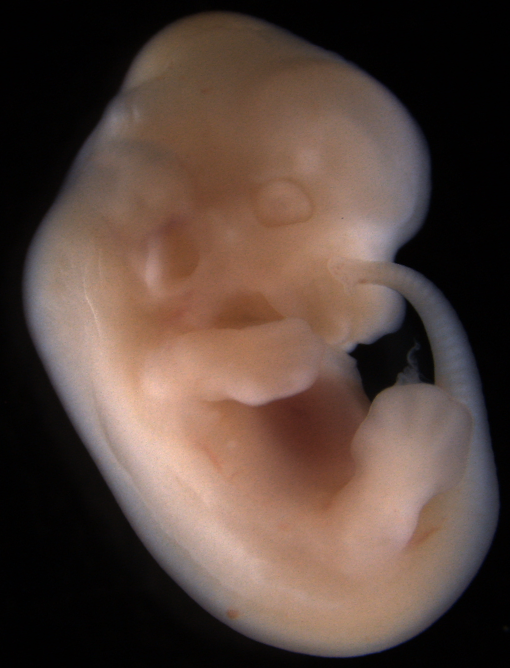

Supplement: Supplementary file 6 — Source data Fig. 1 [file 44319_2024_316_MOESM6_ESM.zip › Soure data_Figure 1/Figure 1B/E12.5/TP39.2(het).tif]

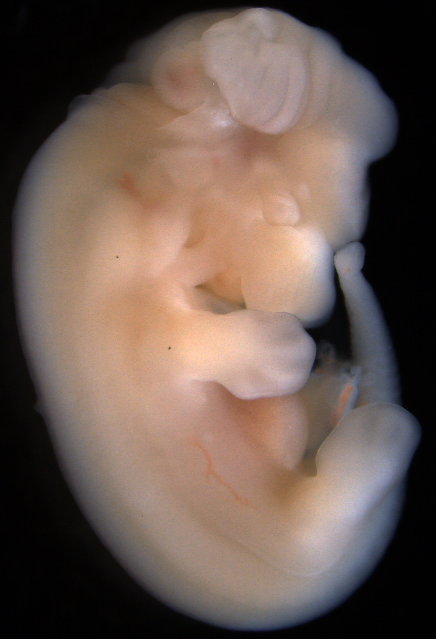

Supplement: Supplementary file 6 — Source data Fig. 1 [file 44319_2024_316_MOESM6_ESM.zip › Soure data_Figure 1/Figure 1B/E12.5/TP39.6(ko).tif]

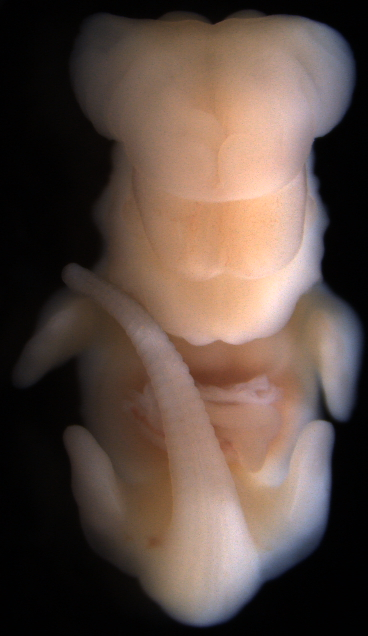

Supplement: Supplementary file 6 — Source data Fig. 1 [file 44319_2024_316_MOESM6_ESM.zip › Soure data_Figure 1/Figure 1B/E12.5/TP39.6(ko)front.tif]

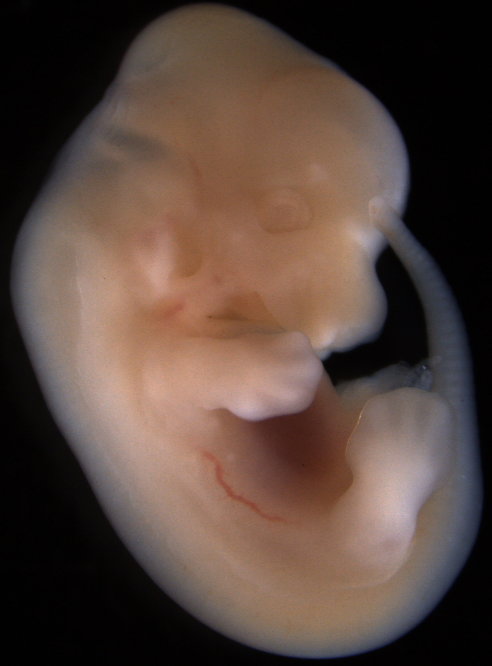

Supplement: Supplementary file 6 — Source data Fig. 1 [file 44319_2024_316_MOESM6_ESM.zip › Soure data_Figure 1/Figure 1B/E12.5/TP39.8(wt).tif]

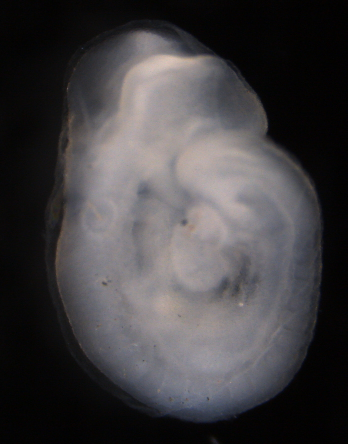

Supplement: Supplementary file 6 — Source data Fig. 1 [file 44319_2024_316_MOESM6_ESM.zip › Soure data_Figure 1/Figure 1B/E9.5/TP15_7(ko).tif]

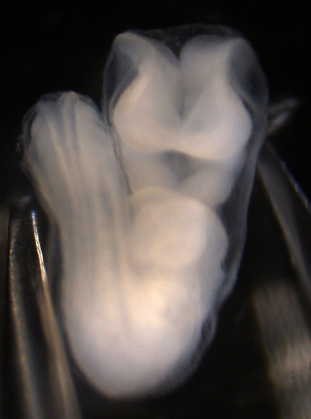

Supplement: Supplementary file 6 — Source data Fig. 1 [file 44319_2024_316_MOESM6_ESM.zip › Soure data_Figure 1/Figure 1B/E9.5/TP15_7(ko)front.tif]

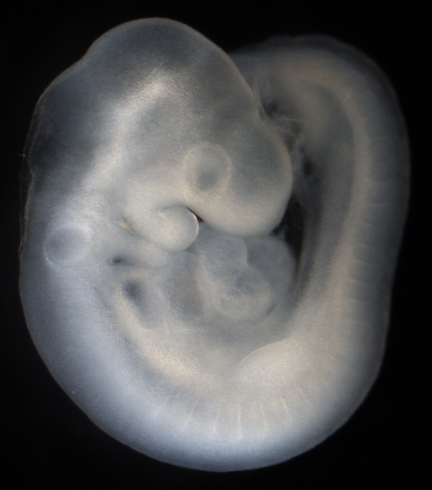

Supplement: Supplementary file 6 — Source data Fig. 1 [file 44319_2024_316_MOESM6_ESM.zip › Soure data_Figure 1/Figure 1B/E9.5/TP15_9(wt).tif]

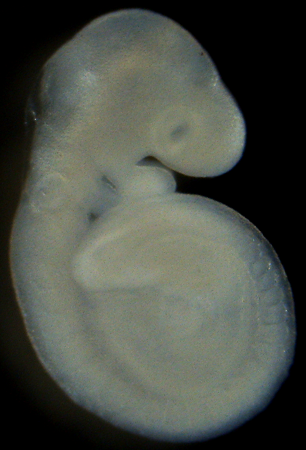

Supplement: Supplementary file 6 — Source data Fig. 1 [file 44319_2024_316_MOESM6_ESM.zip › Soure data_Figure 1/Figure 1B/E9.5/TP27.7(het).tif]

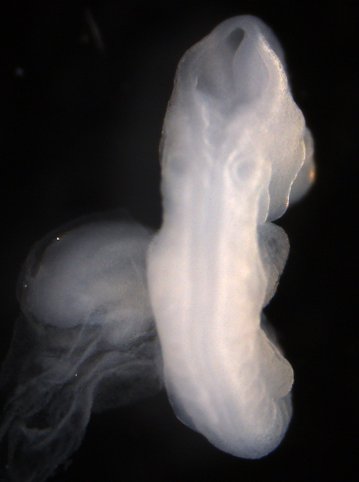

Supplement: Supplementary file 6 — Source data Fig. 1 [file 44319_2024_316_MOESM6_ESM.zip › Soure data_Figure 1/Figure 1C/ko/TP15_11 dorsal.tif]

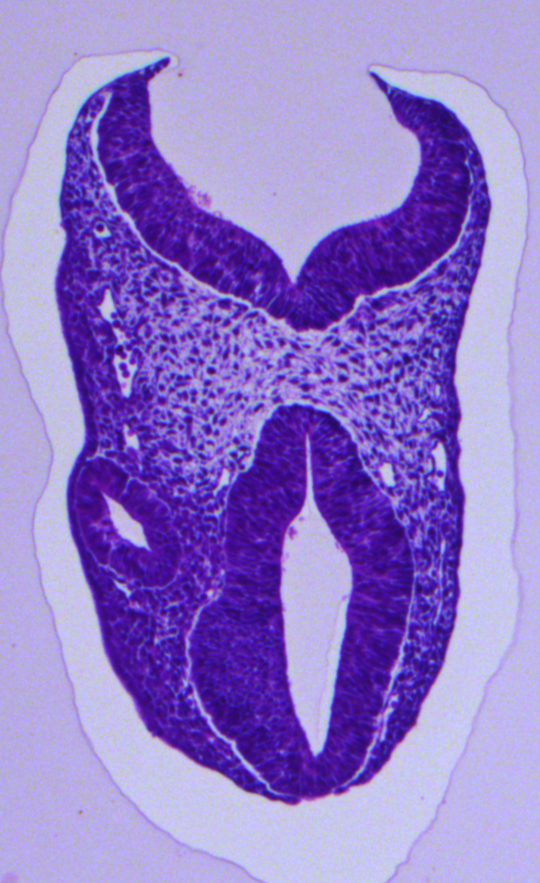

Supplement: Supplementary file 6 — Source data Fig. 1 [file 44319_2024_316_MOESM6_ESM.zip › Soure data_Figure 1/Figure 1C/ko/TP15_11 slide 5 (3).tif]

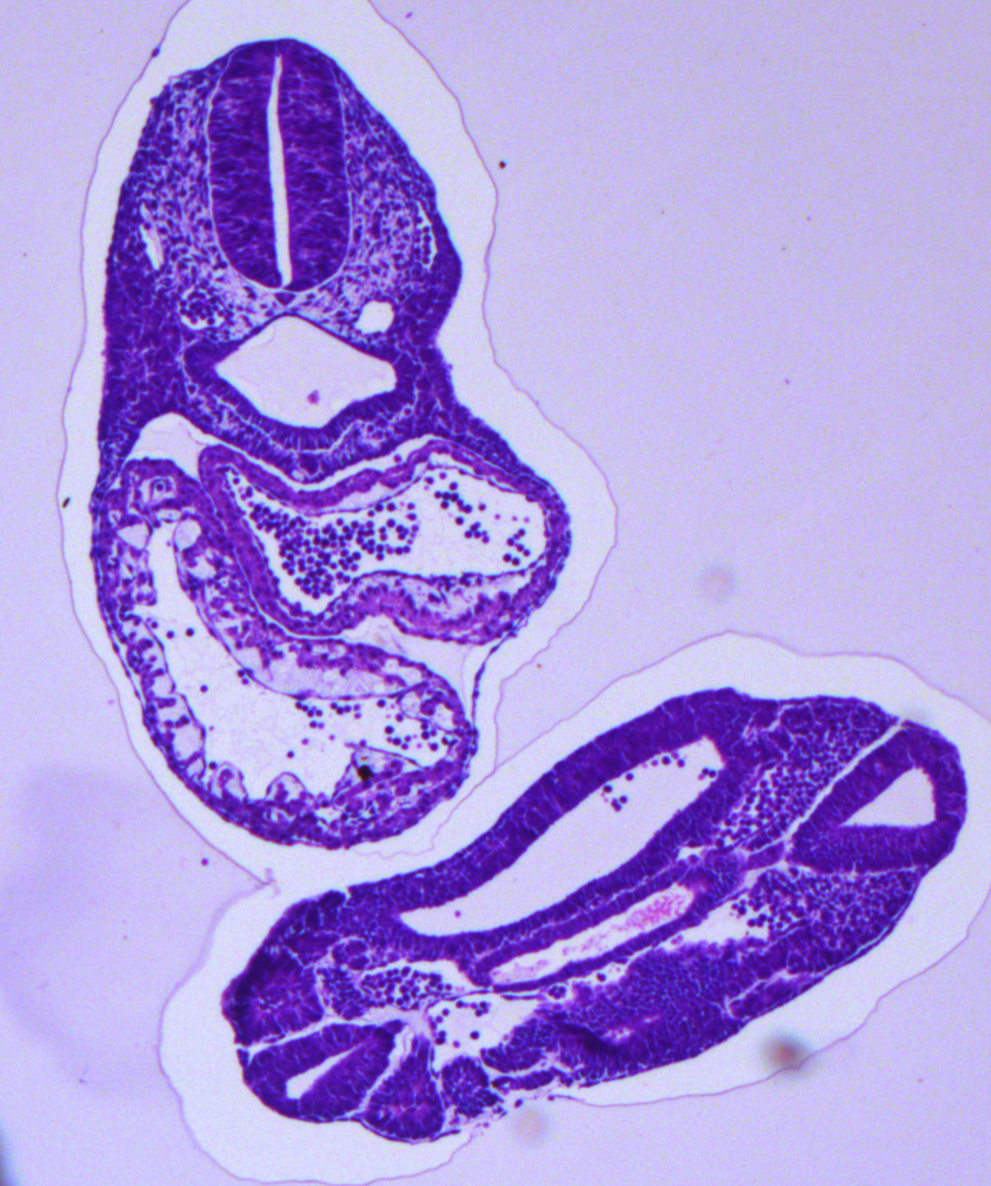

Supplement: Supplementary file 6 — Source data Fig. 1 [file 44319_2024_316_MOESM6_ESM.zip › Soure data_Figure 1/Figure 1C/ko/TP15_11 slide 5 (4).tif]

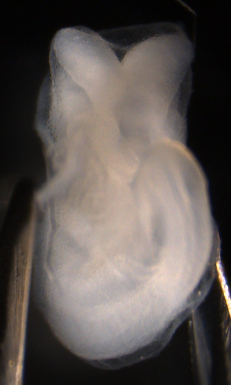

Supplement: Supplementary file 6 — Source data Fig. 1 [file 44319_2024_316_MOESM6_ESM.zip › Soure data_Figure 1/Figure 1C/ko/TP15_8 frontal.tif]

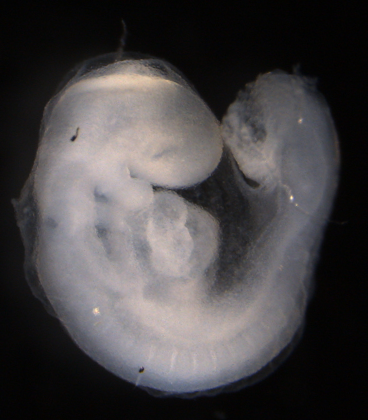

Supplement: Supplementary file 6 — Source data Fig. 1 [file 44319_2024_316_MOESM6_ESM.zip › Soure data_Figure 1/Figure 1C/ko/TP15_8 right.tif]

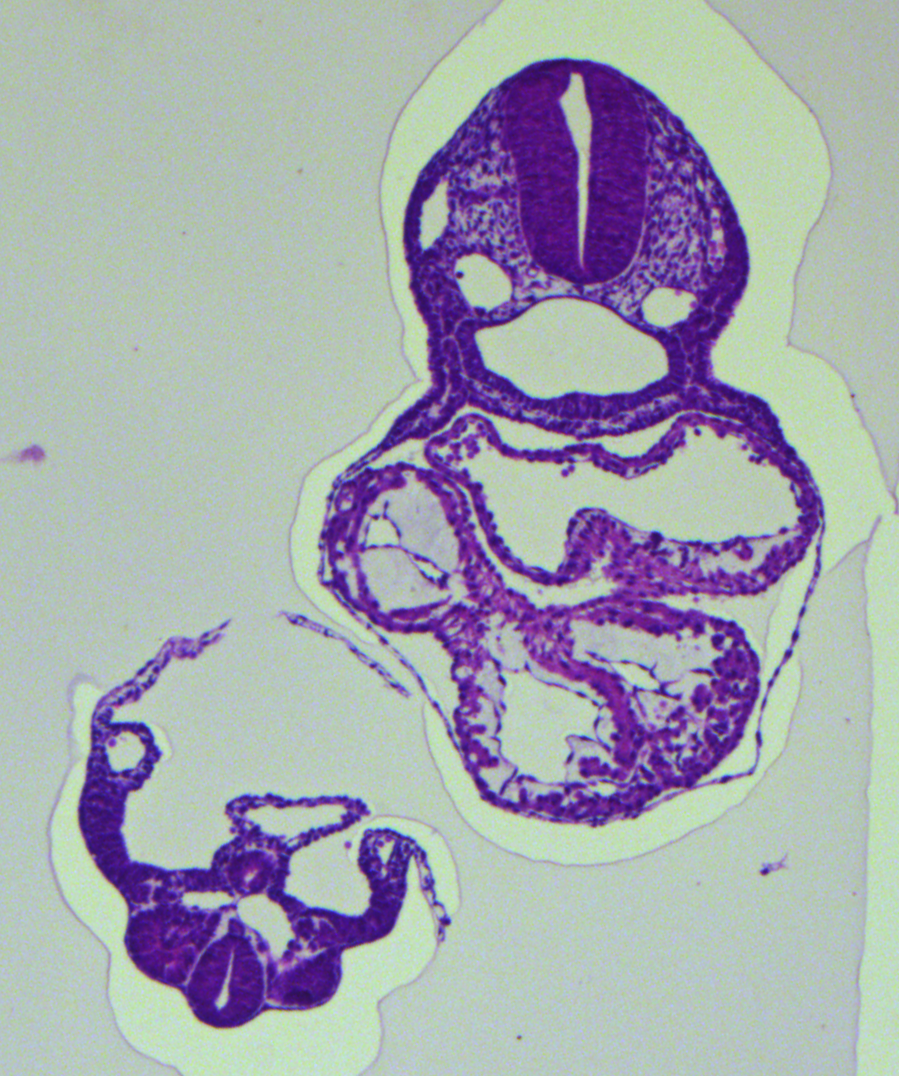

Supplement: Supplementary file 6 — Source data Fig. 1 [file 44319_2024_316_MOESM6_ESM.zip › Soure data_Figure 1/Figure 1C/ko/TP15_8 slide 3 (2).tif]

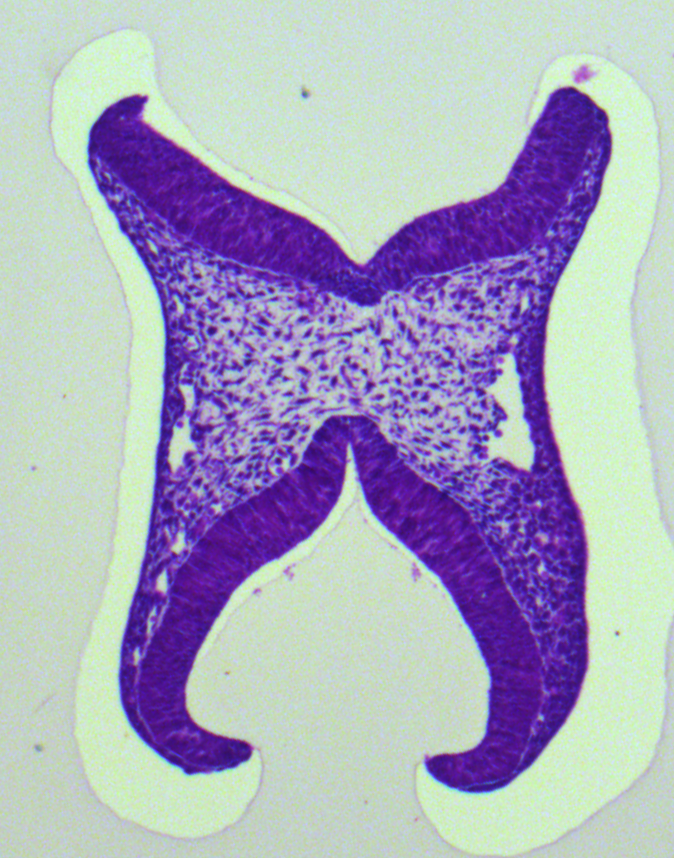

Supplement: Supplementary file 6 — Source data Fig. 1 [file 44319_2024_316_MOESM6_ESM.zip › Soure data_Figure 1/Figure 1C/ko/TP15_8 slide 3 (5).tif]

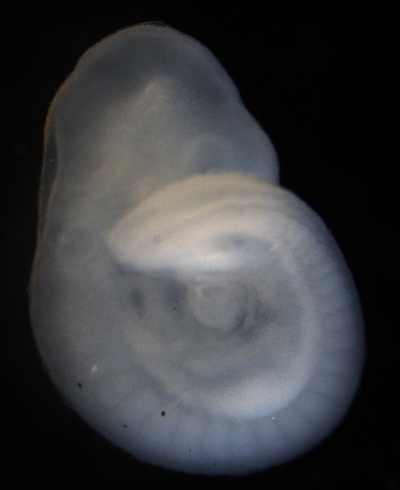

Supplement: Supplementary file 6 — Source data Fig. 1 [file 44319_2024_316_MOESM6_ESM.zip › Soure data_Figure 1/Figure 1C/wt/TP31_3 right anterior wt.tif]

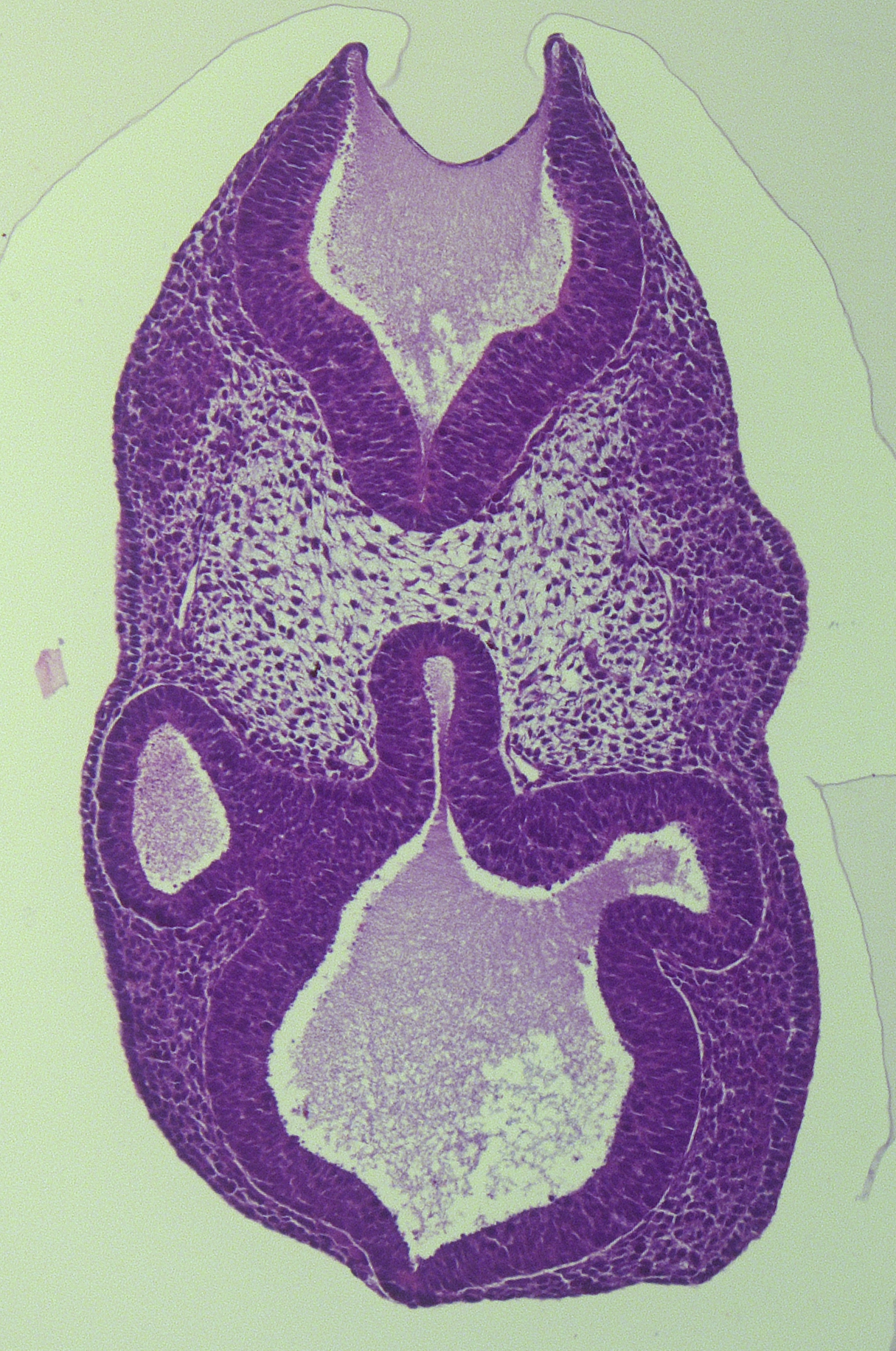

Supplement: Supplementary file 6 — Source data Fig. 1 [file 44319_2024_316_MOESM6_ESM.zip › Soure data_Figure 1/Figure 1C/wt/TP31_3slide2_4(w).tif]

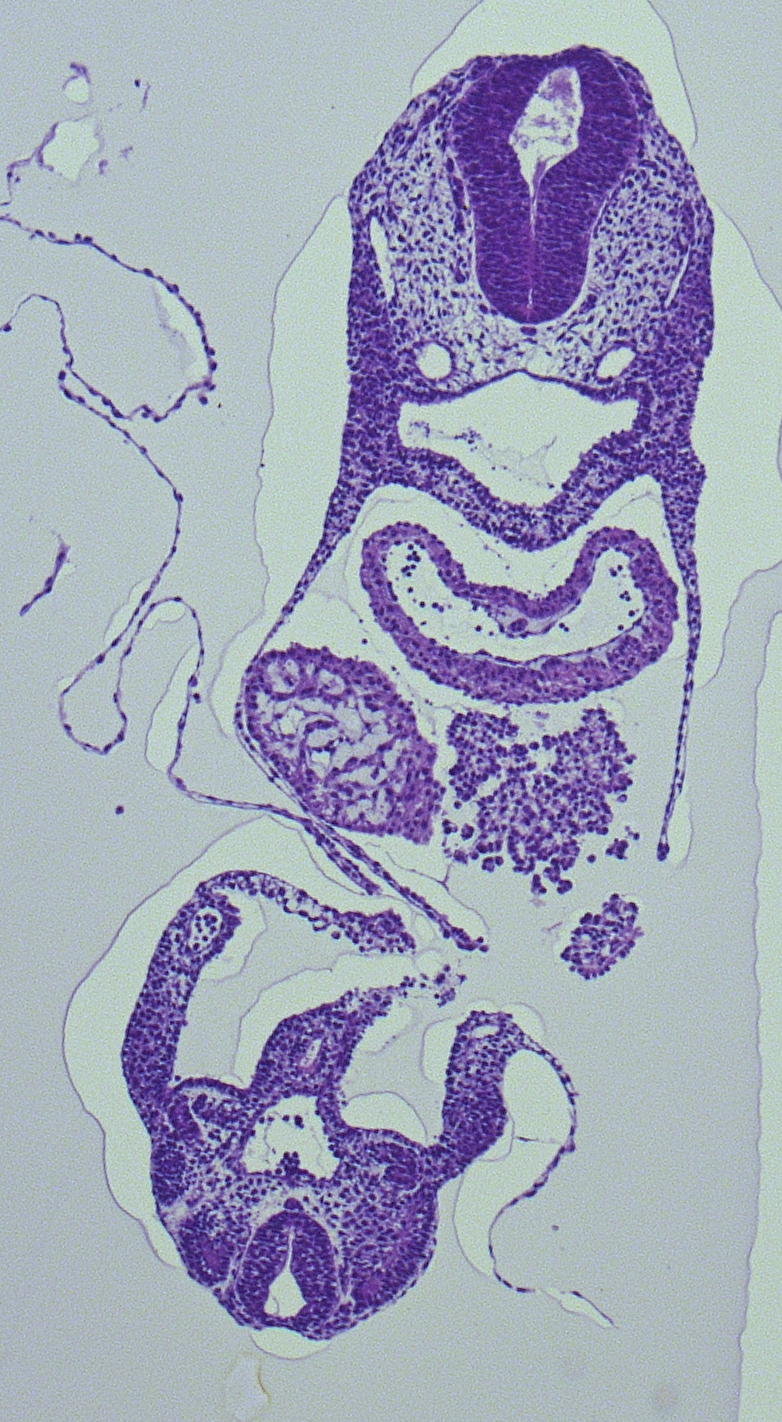

Supplement: Supplementary file 6 — Source data Fig. 1 [file 44319_2024_316_MOESM6_ESM.zip › Soure data_Figure 1/Figure 1C/wt/TP31_3slide2_8(w).tif]

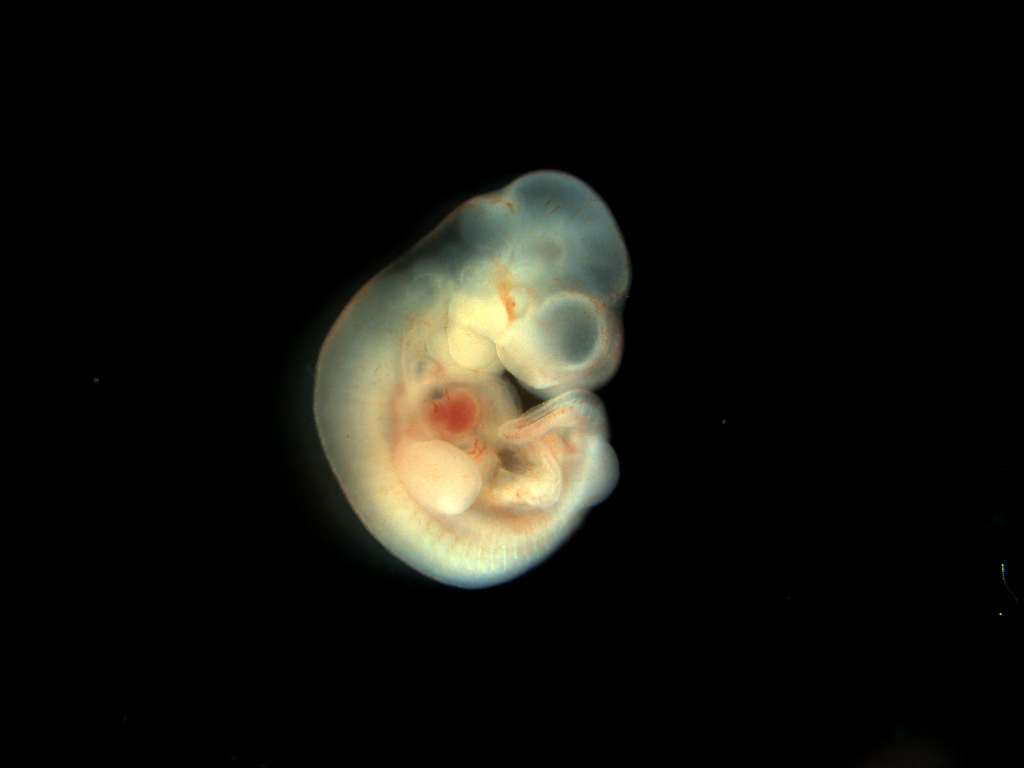

Supplement: Supplementary file 7 — Source data Fig. 2 [file 44319_2024_316_MOESM7_ESM.zip › Soure data_Figure 2/Figure 2D/129FATP54.5-delayed-0.1ppm-ko.tif]

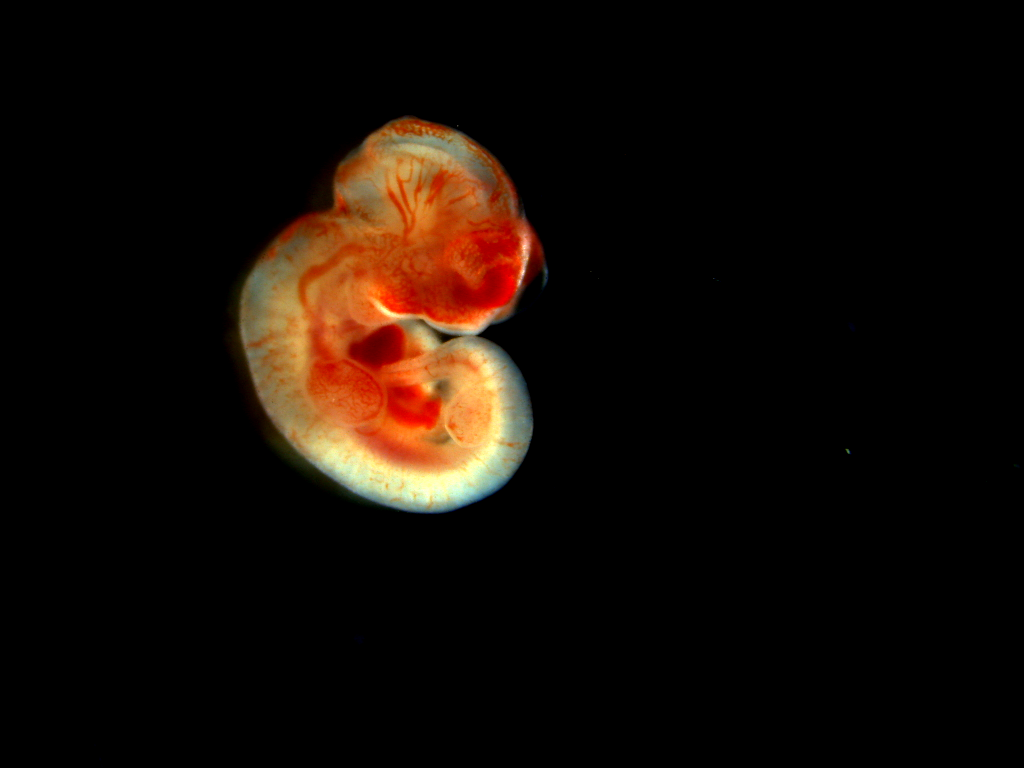

Supplement: Supplementary file 7 — Source data Fig. 2 [file 44319_2024_316_MOESM7_ESM.zip › Soure data_Figure 2/Figure 2D/129S6FA TP16.7-NTD-0.1ppm-het.tif]

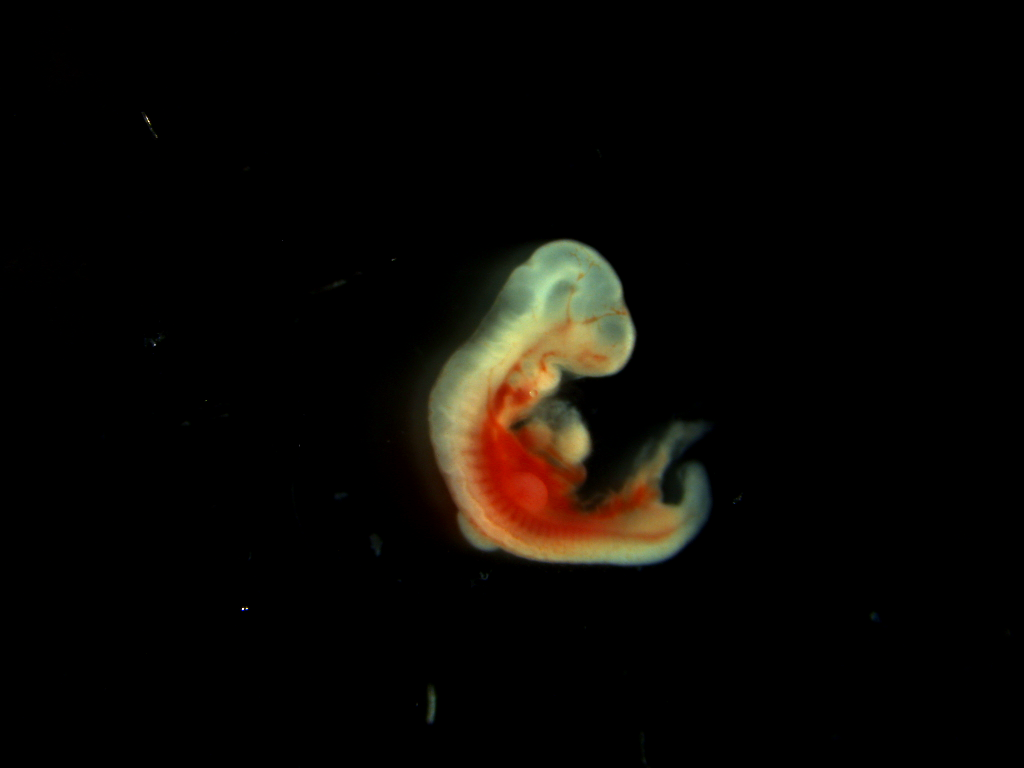

Supplement: Supplementary file 7 — Source data Fig. 2 [file 44319_2024_316_MOESM7_ESM.zip › Soure data_Figure 2/Figure 2D/129S6FA TP35.9-malformed-0.1ppm-ko.tif]

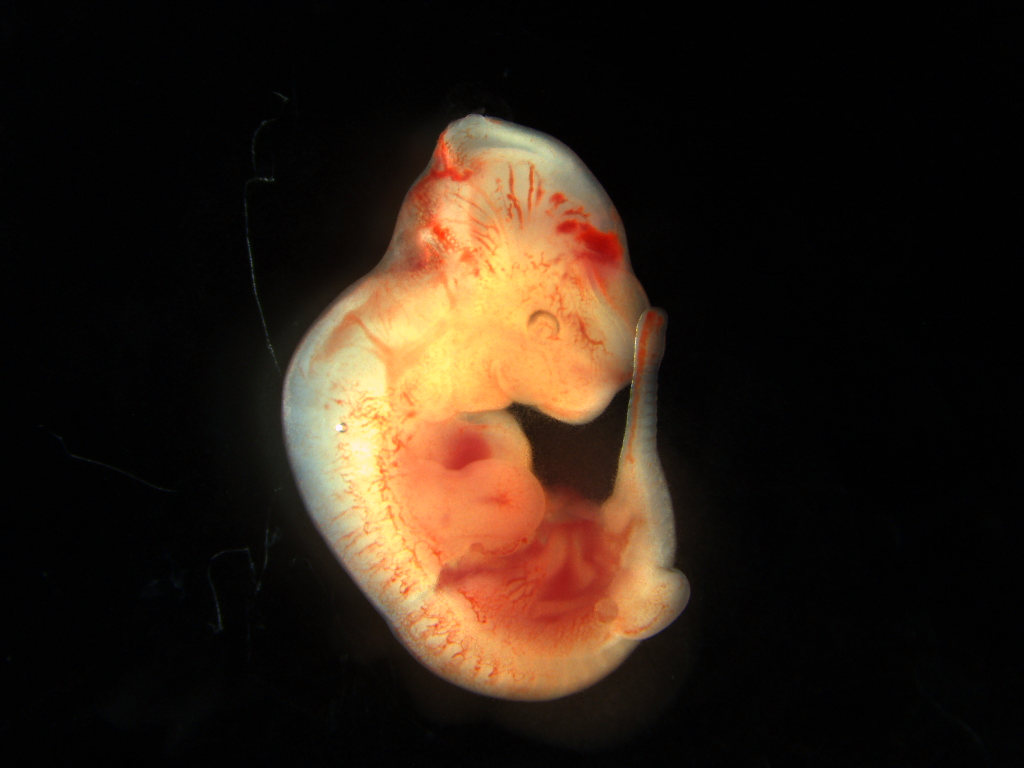

Supplement: Supplementary file 7 — Source data Fig. 2 [file 44319_2024_316_MOESM7_ESM.zip › Soure data_Figure 2/Figure 2D/129S6FA TP50.2-brain malformed severe-0.1-het.tif]

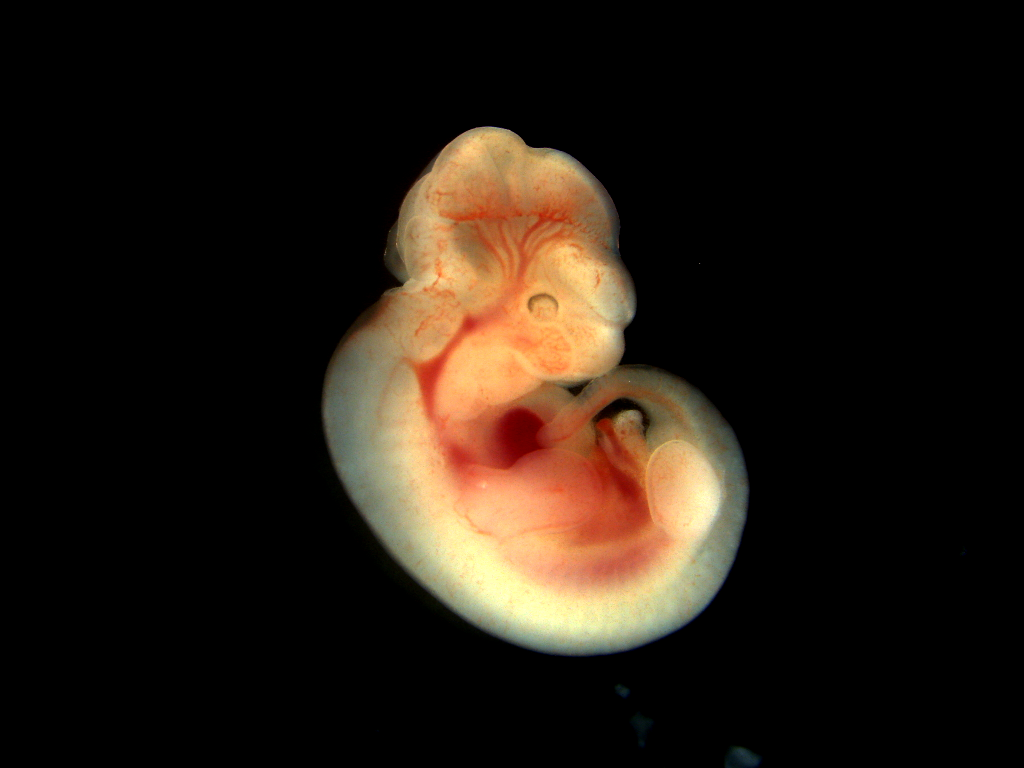

Supplement: Supplementary file 7 — Source data Fig. 2 [file 44319_2024_316_MOESM7_ESM.zip › Soure data_Figure 2/Figure 2D/129S6FA TP57.5-NTD-3ppm-ko.tif]

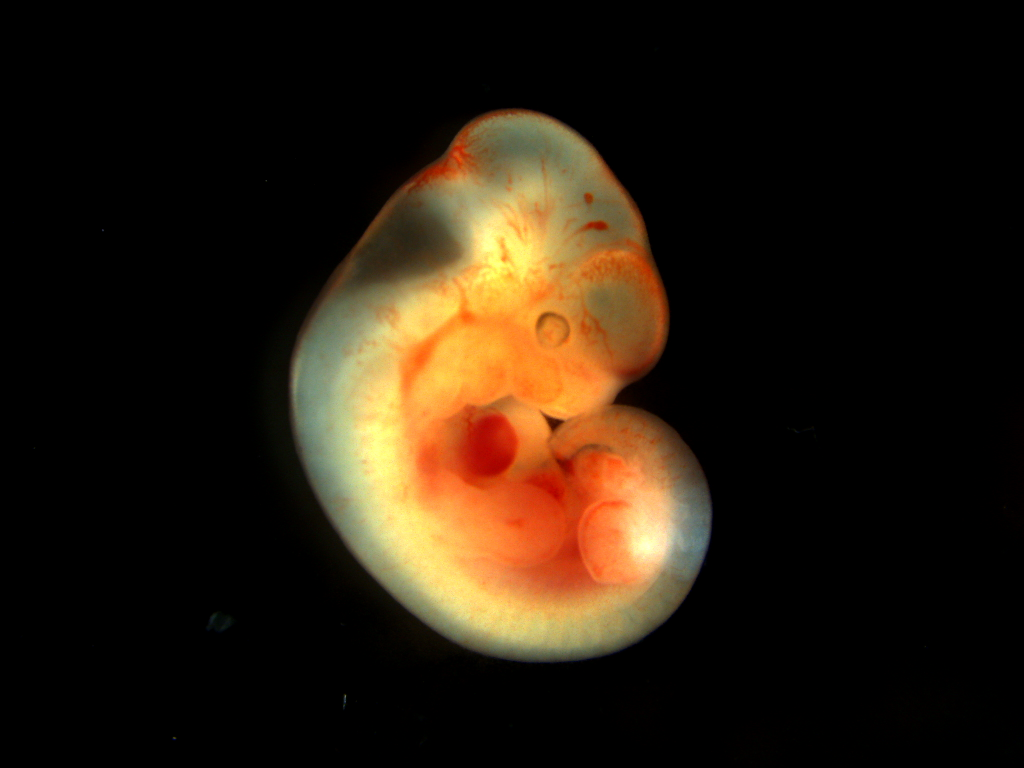

Supplement: Supplementary file 7 — Source data Fig. 2 [file 44319_2024_316_MOESM7_ESM.zip › Soure data_Figure 2/Figure 2D/129S6FA_TP40.3-normal-3ppm-wt.tif]

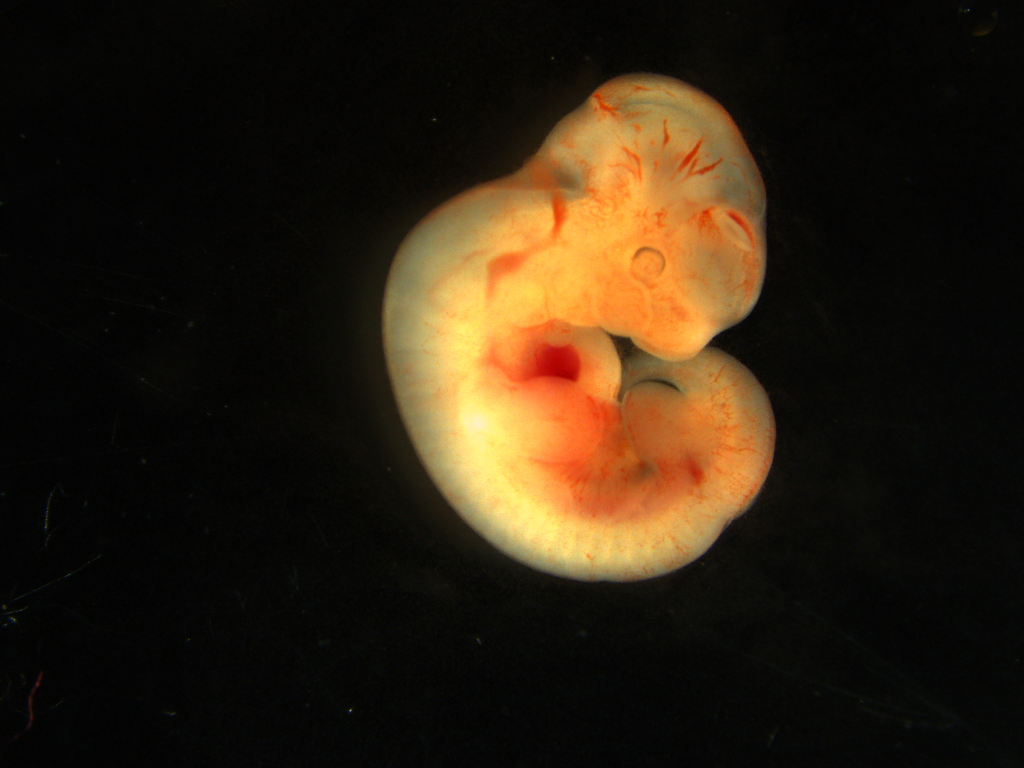

Supplement: Supplementary file 7 — Source data Fig. 2 [file 44319_2024_316_MOESM7_ESM.zip › Soure data_Figure 2/Figure 2D/129S6FA_TP41.7-brain malformed mild-30ppm-het.tif]

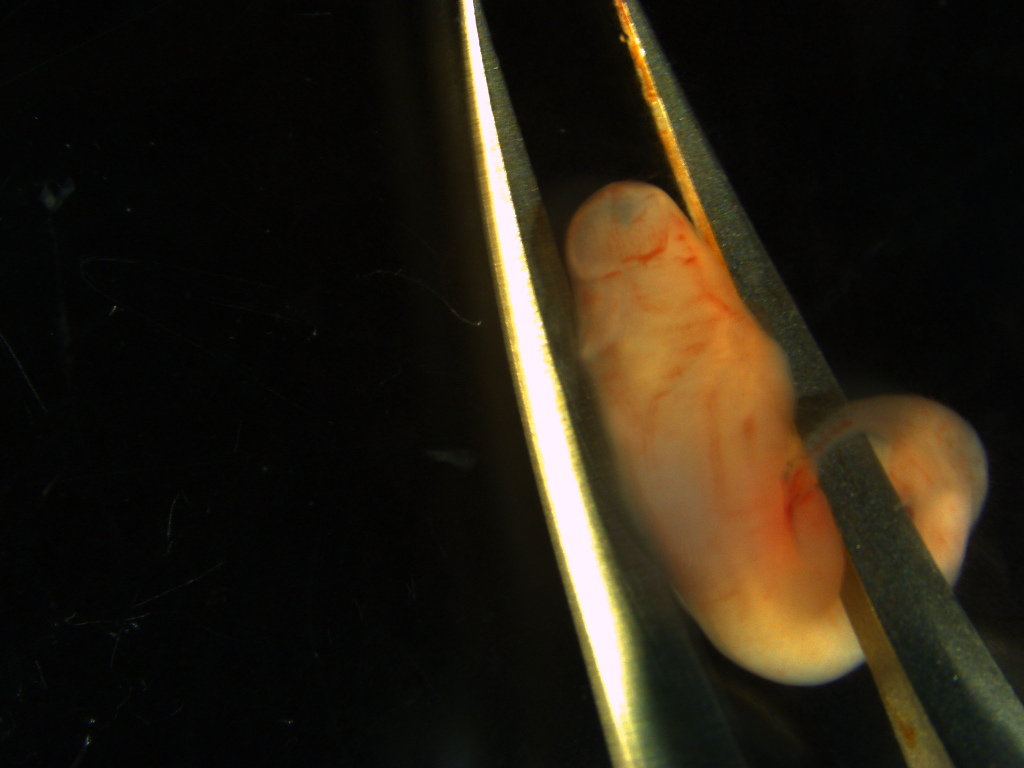

Supplement: Supplementary file 7 — Source data Fig. 2 [file 44319_2024_316_MOESM7_ESM.zip › Soure data_Figure 2/Figure 2D/129S6FA_TP41.7-dorsal.tif]

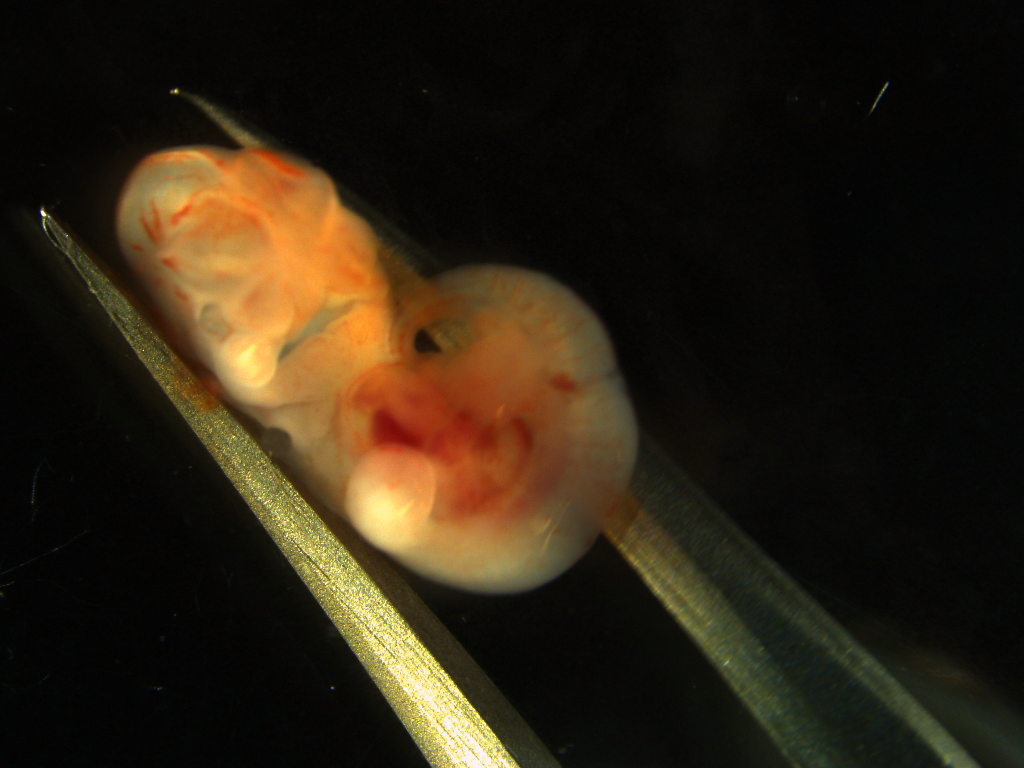

Supplement: Supplementary file 7 — Source data Fig. 2 [file 44319_2024_316_MOESM7_ESM.zip › Soure data_Figure 2/Figure 2D/129S6FA_TP41.7-front.tif]

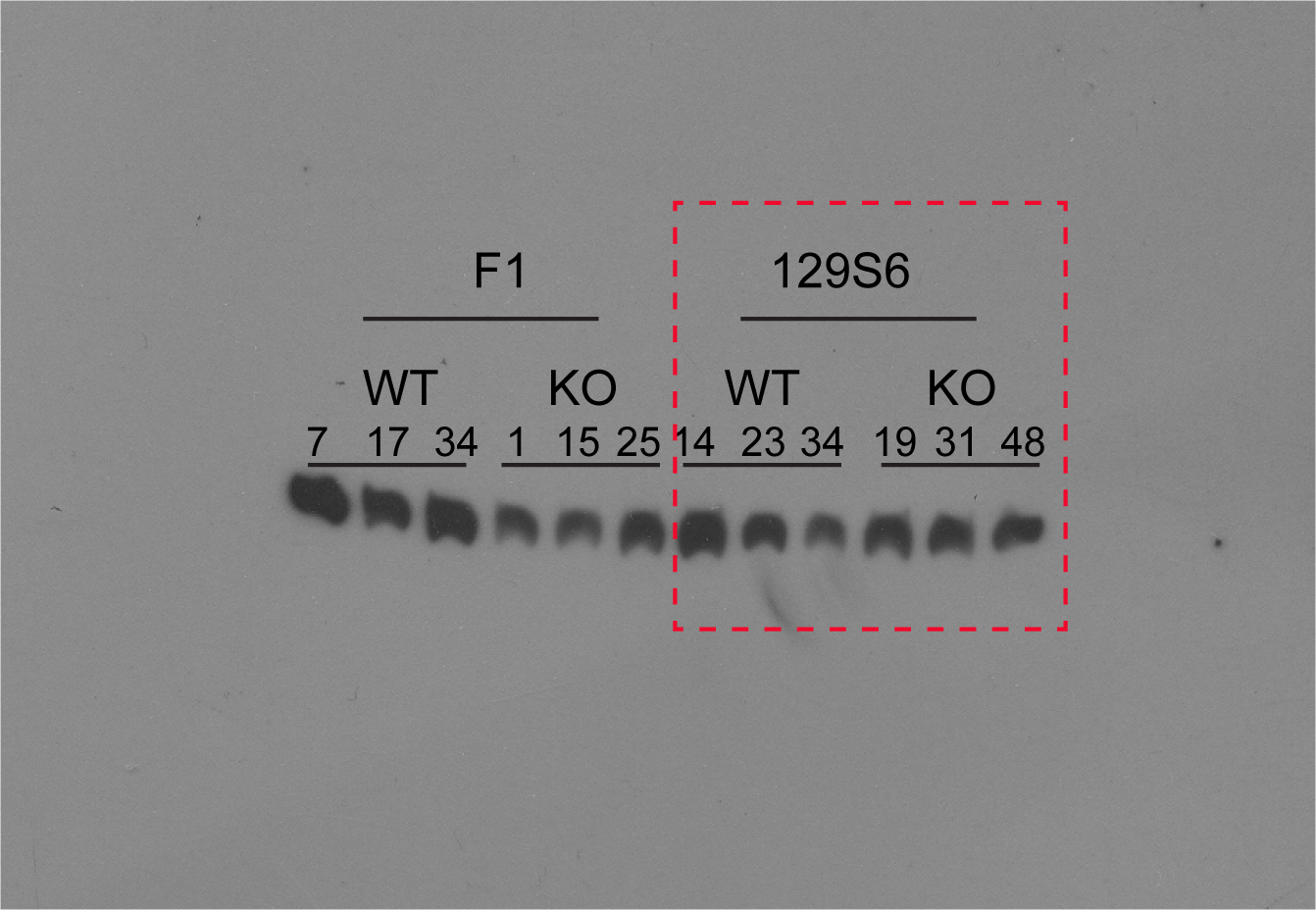

Supplement: Supplementary file 9 — Source data Fig. 6 [file 44319_2024_316_MOESM9_ESM.zip › Soure data_Figure 6/Figure 6D/ACTB-labelled.tif]

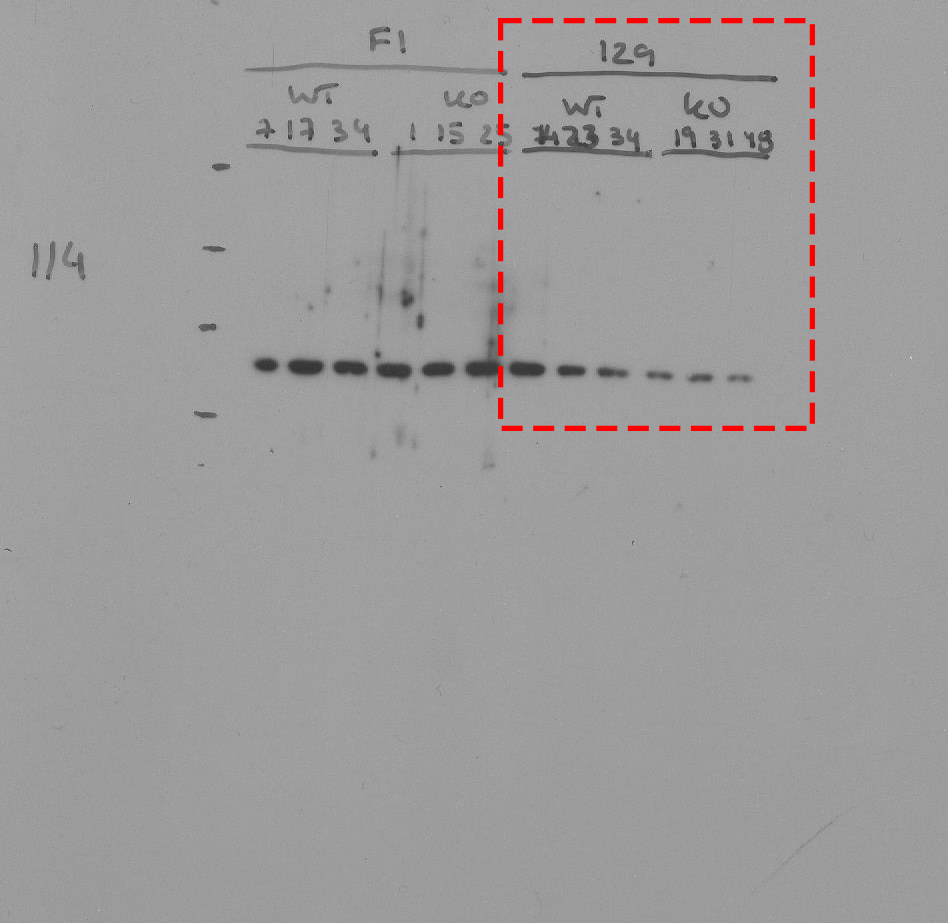

Supplement: Supplementary file 9 — Source data Fig. 6 [file 44319_2024_316_MOESM9_ESM.zip › Soure data_Figure 6/Figure 6D/PCFT-labelled.tif]
